# PPM1H is down-regulated by ATF6 and dephosphorylates p-RPS6KB1 to inhibit progression of hepatocellular carcinoma

**DOI:** 10.1016/j.omtn.2023.06.013

**Published:** 2023-06-19

**Authors:** Xiaoshuang Yang, Jianting Guo, Wei Li, Chunrui Li, Xilin Zhu, Ying Liu, Xiaopan Wu

**Affiliations:** 1 State Key Laboratory of Medical Molecular Biology, Institute of Basic Medical Sciences, Chinese Academy of Medical Sciences, Beijing 100005, P.R. China; 2 School of Basic Medicine, Peking Union Medical College, Beijing 100005, P.R. China; 3 Department of Interventional Radiology, Affiliated Hospital of Qingdao University, Shandong 266003, P.R. China; 4 Beijing Cloud Computing Key Technique and Application Key Laboratory, Beijing Computing Center, Beijing 100094, P.R. China

**Keywords:** MT: RNA/DNA Editing, ATF6, protein phosphatase 1H, RPS6KB1, homology modeling, hepatocellular carcinoma

## Abstract

We have shown previously that polymorphism of activating transcription factor 6 (ATF6) is associated with susceptibility to hepatocellular carcinoma (HCC). Therefore, genes down-regulated by ATF6 might play a tumor-suppressing role. In the present study, we identified that expression of protein phosphatase magnesium- or manganous-dependent 1H (PPM1H) mRNA and protein can be inhibited by ATF6 in hepatoma cells and mice with liver *Atf6* knockdown. Tumor tissues from 134 HCC patients were analyzed by immunohistochemistry, and PPM1H exhibited higher expression levels in adjacent para-cancer tissues than in HCC tissues. Therefore, patients with higher expression of PPM1H had a better prognosis. PPM1H inhibited proliferation, migration, and invasion of hepatoma cells. In addition, PPM1H inhibited induced HCC nodule formation as well as tumor xenograft growth in diethylnitrosamine/CCl_4_-induced HCC mouse model and nude mouse tumorigenicity assay, respectively. A 3D model of PPM1H was obtained by homology multi-template modeling, and ribosomal protein S6 kinase B1 (RPS6KB1) in the bone morphogenetic protein (BMP)/transforming growth factor β (TGF-β) pathway was screened out as the potential substrate of PPM1H by Rosetta. PPM1H could directly dephosphorylate p-RPS6KB1. To conclude, we discovered RPS6KB1 as a new PPM1H dephosphorylation substrate. PPM1H exhibited a suppressive effect on HCC progression by dephosphorylating p-RPS6KB1.

## Graphical abstract

**Figure:**
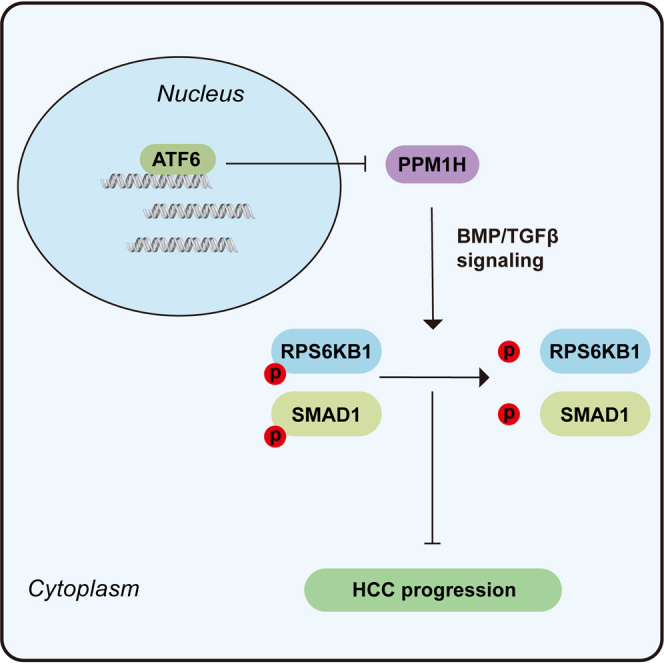

## Teaser

This study found that PPM1H inhibits proliferation, migration, and invasion of hepatoma cells *in vitro* and suppresses HCC progression *in vivo* and identified PPM1H as the phosphatase of RPS6KB1 to regulate HCC development. PPM1H expression can be inhibited by ATF6. The ATF6-PPM1H-RPS6KB1 pathway might provide a potential HCC therapeutic target.

## Introduction

Currently, liver cancer is one of the most common malignant tumors, causing about 841,000 new cases and 782,000 deaths in the world every year according to the Global Cancer Observatory (GLOBOCAN) in 2018.^1^ Thus, liver cancer ranks fifth in terms of global cases and second in terms of deaths for males. Among them, 55% of the patients are of Chinese origin. Hepatocellular carcinoma (HCC) is the most common type of liver cancer and comprises 75–85% of cases. Other liver cancer types include intrahepatic cholangiocarcinoma (comprising 10%–15% of cases) as well as other rare types.^1^ Because of the lack of reliable early diagnostic markers and effective treatments, clinical treatment of liver cancer is still not satisfactory.^2^^,^^3^ Therefore, exploring the pathogenesis of HCC and developing markers related to early diagnosis and prognosis are becoming increasingly important for new and more effective HCC treatment modalities.

The endoplasmic reticulum (ER) is a common feature of eukaryotic cells and plays an important role in protein folding, processing, and transport.^4^^,^^5^ ER homeostasis can be destroyed by numerous stimuli, like oxidative stress, nutrient fluctuation, calcium ion imbalance, viral infection, and more, leading to an accumulation of unfolded or misfolded proteins that trigger ER stress, which results in activation of the unfolded protein response (UPR) signaling pathway.^6^^,^^7^ Activating transcription factor 6 (ATF6) is one of the key stress sensors on the ER membrane. When the UPR is activated, ATF6 transfers to the Golgi apparatus and is cleaved to form activated ATF6, regulating transcription and expression of downstream genes to help cells adapt to ER stress.^8^^,^^9^^,^^10^ ATF6 plays a vital role in a variety of cancers; e.g., by regulating cell growth, migration, apoptosis, and autophagy in cervical cancer.^11^ ATF6-associated autophagy participates in ovarian cancer cell chemoresistance,^12^ death-associated protein kinase 1 (DAPK1)-related chronic lymphocytic leukemia cell death,^13^ and cancer-associated fibroblast (CAF) activation in lung adenocarcinoma progression.^14^ In colorectal cancer, ATF6 induces intestinal dysbiosis and tumorigenesis.^15^ High ATF6 expression is correlated with a poor prognosis in colon cancer.^16^ Several studies have confirmed that, in human HCC, expression of ATF6 is upregulated.^17^^,^^18^ Furthermore, in our previous studies we found that polymorphism of ATF6 is associated with susceptibility to HCC^19^ as well as promotion of HCC progression.^20^ Through RNA sequencing (RNA-seq) in Hep-G2 cells (NCBI: SAMN11835389),^20^ cystathionine-γ-synthase (CTH) has been identified as a downstream gene of ATF6 that enhances production of H_2_S in hepatoma cells, leading to promotion of HCC development. We also established a mouse model in previous studies.^20^ The transgenic *Atf6*^*fl/fl*^ C57BL/6 mice purchased from The Jackson Laboratory possessed *loxP* sites flanking exons 8 and 9 of the *Atf6* gene*.* They hybridized with Alb-cre C57BL/6 mice and generated *Atf6*^*Δhep*^ offspring mice, which are liver-specifically *Atf6* deficient compared with the *Atf6*^*fl/fl*^ mice. Because of the tumor-promoting role of ATF6, genes down-regulated by ATF6 might play a tumor-suppressing role in HCC development. Therefore, in the present study, we aimed to identify downstream genes that are downregulated by ATF6 and clarify their role in HCC progression.

## Results

### ATF6 inhibits expression of PPM1H

**Figure 1:**
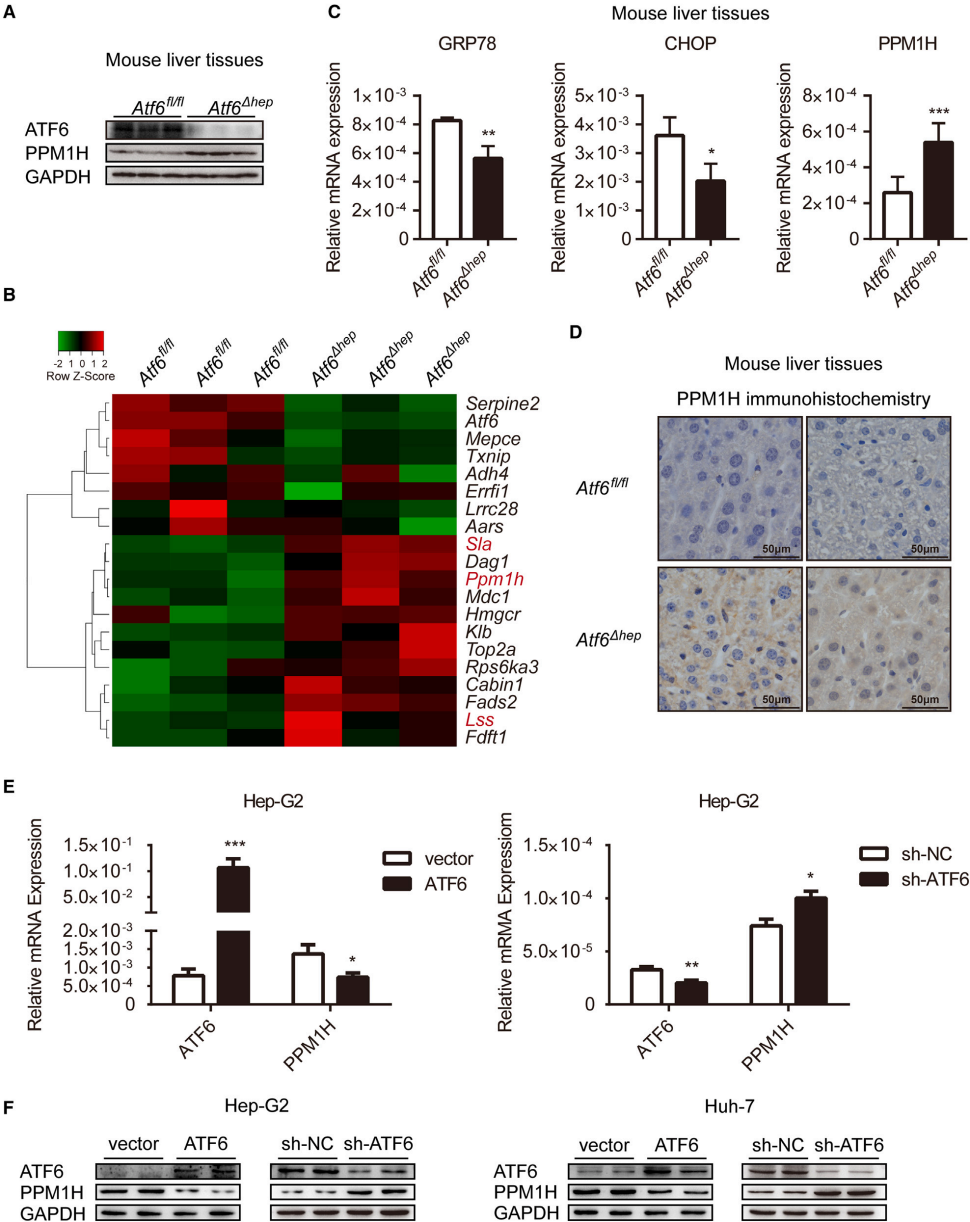
ATF6 inhibits expression of PPM1H in hepatoma cells (A) Western blot analysis of ATF6 and PPM1H protein expression in mouse liver tissues. (B) Heatmap of the differentially expressed genes filtered out according to RNA-seq in Hep-G2 cells and mice. (C) Quantitative real-time PCR analyzed the mRNA expression of GRP78, CHOP, and PPM1H in mouse liver tissues. (D) IHC assay of PPM1H in the liver tissues of *Atf6*^*fl/fl*^ and *Atf6*^*Δhep*^ mice. Magnification, ×400. (E and F) Vector, ATF6, sh-NC, or sh-ATF6 plasmids were transfected into Hep-G2 or Huh-7 cells to overexpress or knock down ATF6. NC, negative control. (E) Quantitative real-time PCR analyzed the mRNA level of ATF6 and PPM1H in Hep-G2 cells; data were normalized to glyceraldehyde-3-phosphate dehydrogenase (GAPDH). (F) Western blot analysis of ATF6 and PPM1H protein expression in Hep-G2 and Huh-7 cells. Data represent the mean ± SD of three independent experiments. ∗p < 0.05, ∗∗p < 0.01, ∗∗∗p < 0.001.

As a key effector of the adaptive UPR pathway, ATF6 can increase the volume of the ER and reduce synthesis of new proteins, resulting in promotion of cell survival. In this way, ATF6 plays a cancer-promoting role in colorectal cancer, prostate cancer, and other cancers.^21^^,^^22^ In ATF6-overexpressing Hep-G2 cells, the volume of the ER expanded significantly (Figure S1A), which is consistent with the observation of adaptive UPR responses in ER stress. In this study, we first tried to identify downstream genes negatively regulated by ATF6. Based on RNA-seq data (NCBI: SAMN11835389) of Hep-G2 cells,^20^ 140 transcripts with a significant difference (p-adjusted value, padj < 0.001) in expression were screened. To further verify these results in Hep-G2 cells, we conducted a new RNA-seq analysis in *Atf6*^*fl/fl*^ and *Atf6*^*Δhep*^ mice (NCBI: PRJNA752835). Details of the sequencing and analyzing programs (such as HISAT2 alignment, Genome Analysis Toolkit, etc.) were conducted as described under Materials and Methods. The sequencing coverage and quality statistics of the genomic next-generation sequencing generated in this study are summarized in Table S1. Western blot assays revealed that ATF6 expression was knocked down in livers of *Atf6*^*Δhep*^ mice (Figure 1A). Then, we performed a cross-over analysis of the RNA-seq results in cells and mouse liver tissues of the 140 transcripts screened in Hep-G2 cells. Of these, 107 overlapped with those of mouse liver tissues, and 91 of them exhibited the same regulation trend. The top 20 genes with the most significant differences are listed in the heatmap in Figure 1B. We chose the top 3 down-regulated genes (*Lss*, *Sla*, and *Ppm1h*) and examined their mRNA expression in cells and mouse liver tissues. The relative mRNA expression levels of the three genes of interest in ATF6-overexpressing cells and in *Atf6*^*Δhep*^ mice were validated by quantitative real-time PCR, with PPM1H showing the most pronounced differentiation (Figure S1B). Western blot, quantitative real-time PCR, and immunohistochemistry assays were performed in mice to further confirm this. Consistently, the expression level of PPM1H mRNA and protein markedly increased in *Atf6*^*Δhep*^ mice (Figures 1A, 1C, and 1D). The mRNA expression levels of the ATF6 downstream genes GRP78 and CHOP were notably impaired in *Atf6*^*Δhep*^ mice, which is consistent with previous studies.^23^ The PPM1H protein expression levels in liver tissues of full *Atf6* knockout (*Atf6*^*KO*^) mice were detected and showed the same trend as in *Atf6*^*Δhep*^ mice (Figure S1C). Hep-G2 and Huh-7 cell lines were used to further verify the negative regulation of ATF6 and PPM1H. We constructed ATF6 overexpression plasmids and shATF6 plasmids expressing short hairpin RNA against ATF6. These plasmids were transfected into cells to overexpress or silence ATF6 expression. As expected, the expression levels of PPM1H mRNA and protein were obviously impaired in Hep-G2 and Huh-7 cells overexpressing ATF6. In contrast, knockdown of ATF6 by shATF6 increased the mRNA and protein expression of PPM1H (Figures 1E and 1F), which suggests that ATF6 was able to suppress the expression of PPM1H. Previous studies have shown that PPM1H is mainly localized in the cytoplasm, especially in the Golgi apparatus. In the results of the western blots, PPM1H showed no ER localization (Figure S1D).

To further validate the inhibition of ATF6 on PPM1H expression, we designed a recombinant fusion protein consisting of ATF6 fused with protein transduction domain of *trans* activator of transcription (PTD) (Figure S1E), the latter being a cell-penetrating peptide.^24^ When the cells were incubated with different concentrations of PTD-ATF6, we observed that, after treatment with 30 μg/mL recombinant protein, the ATF6 level in the cells was higher than that of the other groups (Figure S1F). Moreover, at this concentration, the fusion protein also promoted the proliferation of cancer cells, which could also be observed after transfection experiments with FLAG-ATF6 (Figure S1G). This result indicates that *in vitro*-synthesized ATF6 was able to enter the cells with the help of the PTD domain and affected proliferation downstream. In addition, 30 μg/mL PTD-ATF6 inhibited expression of PPM1H in Hep-G2 cells (Figure S1H). Moreover, treatment with PTD-ATF6 could promote expression of the mRNA of GRP78 and CHOP and reduced expression of PPM1H (Figure S1I), which further confirms that ATF6 was able to inhibit expression of PPM1H. Taken together, these data show that the mRNA and protein expression of PPM1H is downregulated by ATF6 *in vitro* and *in vivo*.

To explore how ATF6 inhibits expression of PPM1H, three PPM1H promoter-PGL3 plasmids containing PPM1H 5′ UTR sequences and full-length (pro-full) and truncated (pro-716) promoter sequences were constructed to perform a dual-luciferase assay in Hep-G2 cells (Figure S2A). However, the luciferase activities exhibited no significant differences when ATF6 was overexpressed in the three groups (Figure S2B), showing that ATF6 inhibited PPM1H expression not through transcriptional ways. Actinomycin D was added to the culture medium of Hep-G2 and Huh-7 cells to investigate the degradation rate of PPM1H mRNA. ATF6 overexpression was validated at each time point using western blotting. However, no significant difference was shown between cells with ATF6 overexpression and the control group (Figure S2C). These results indicate that ATF6 had almost no influence on promoter activity or PPM1H mRNA degradation. ATF6-overexpressing Hep-G2 and Huh-7 cells were treated with the proteasome inhibitor MG132, and the protein expression level of ATF6 increased with prolonged treatment time in vector- and ATF6-transfected cells as well as PPM1H, suggesting that the stability of PPM1H protein may not be affected by ATF6 (Figure S2D). Plasmid ATF6(1–7) contained the coding sequence of ATF6 exons 1–7 and expressed a mutant ATF6 protein that is able to interact with other proteins but unable to bind to DNA. Interestingly, ATF6(1–7) obviously inhibited PPM1H mRNA expression (Figure S2E), indicating that ATF6 might play a suppressing role through interaction with other proteins; however, the exact mechanism needs to be determined.

### PPM1H mediates proliferation and metastasis of HCC cells

**Figure 2:**
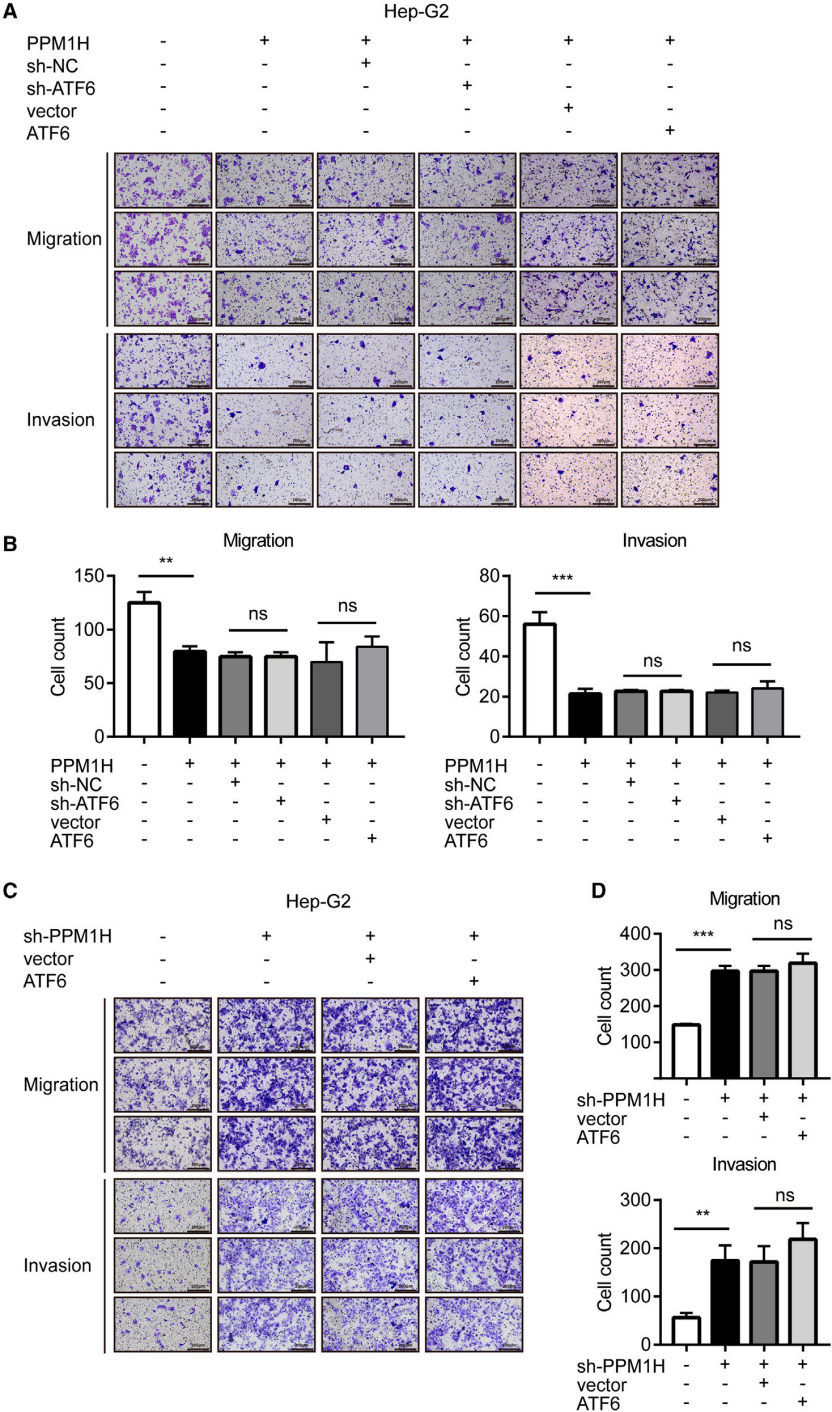
PPM1H inhibited migration and invasion of hepatocellular carcinoma cells (A and C) Transwell assays revealed Hep-G2 cell migration and invasion after transfection of the indicated plasmids. (B and D) Quantification of (A) and (C), respectively. Data represent the mean ± SD of three independent experiments. ∗p < 0.05, ∗∗p < 0.01, ∗∗∗p < 0.001. Magnification, ×100.

Methyl thiazolyl tetrazolium (MTT) assays were conducted to evaluate the activity of hepatoma cells. The results showed that the proliferation of Hep-G2 and Huh-7 cells decreased when PPM1H was overexpressed, while it was increased when PPM1H was inhibited (Figure S3A). Soft agar assays were performed to evaluate the anchorage-independent growth of cells, which is a feature of malignant proliferation. Consistently, PPM1H overexpression inhibited the colony formation rate of Hep-G2 and Huh-7 cells, while knockdown of PPM1H promoted colony formation of cells (Figures S3B and S3C). On the other hand, PPM1H inhibited the migration and invasion of Hep-G2 and Huh-7 cells (Figures 2A, 2B, S4A, and S4B). Moreover, knockdown of PPM1H enhanced the migration and invasion of hepatoma cells (Figures 2C, 2D, S4C, and S4D). However, there was no significant difference in the migration and invasion activity of PPM1H-overexpressing hepatoma cells regardless of whether ATF6 was overexpressed or knocked down (Figures 2A, 2B, S4A, and S4B). Consistently, in PPM1H knockdown cells, co-transfecting ATF6 plasmids barely altered the migration and invasion of Hep-G2 and Huh-7 cells (Figures 2C, 2D, S4C, and S4D), indicating that ATF6 regulates the proliferation and metastasis of hepatoma cells through the function of PPM1H. In general, PPM1H, as a downstream gene of ATF6, has a suppressive effect on the growth of hepatoma cells.

As ATF6 downstream genes, GRP78 and CHOP play an essential role in regulation of the UPR. The mRNA expression levels of GRP78 and CHOP are downregulated in liver tissues of *Atf6*^*Δhep*^ mice, as shown in Figure 1C. However, the mRNA expression levels of GRP78 and CHOP hardly changed in PPM1H-overexpressing Hep-G2 and Huh-7 cells (Figures S5A and S5B), indicating that PPM1H regulated the progression of HCC in a GRP78- and CHOP-independent manner.

Migration and invasion of epithelium-derived malignant cells is associated with epithelial-mesenchymal transition (EMT). It manifests decreases in E-cadherin and ZO-1 expression, which are characteristics of epithelial cells, and an increase in expression of the mesenchymal markers N-cadherin and vimentin. Western blot analysis of the EMT markers showed that PPM1H can promote E-cadherin and ZO-1 expression and inhibit expression of N-cadherin and vimentin (Figures S5C and S5D). Conversely, inhibiting PPM1H expression resulted in decreasing E-cadherin and ZO-1 and increasing N-cadherin and vimentin expression (Figures S5C and S5D). Thus, PPM1H might inhibit the migration and invasion of hepatoma cells by affecting the EMT characteristics of cancer cells.

PPM1H is a specific phosphatase of the drosophila mothers against decapentaplegic protein SMAD1/5/8 complex,^25^ which plays a role in regulating HCC progression as the downstream factor of BMP.^26^ ALK3 (Q233D) mutation of BMPR1A is able to continuously phosphorylate SMAD1. In this study, Hep-G2 cells were co-transfected with FLAG-SMAD1, BMPR1A(ALK3Q233D), and LV-PPM1H. The results of these experiments showed that the phosphorylation level of SMAD1 in hepatoma cells was markedly decreased under the influence of PPM1H (Figure S5E). The BMP type I receptor inhibitor LDN193189 effectively inhibited phosphorylation of p-SMAD under the experimental conditions used.^27^ Interestingly, overexpressing PPM1H still inhibited Transwell migration and invasion of Hep-G2 and Huh-7 cells after blocking phosphorylation of SMAD1 with LDN193189 (Figure S5F), pointing to the fact that there might be other substrates of PPM1H that regulate hepatoma cell growth and invasion. Therefore, it is necessary to search for new PPM1H substrates.

### Computer-simulated protein docking experiments predict ribosomal protein S6 kinase B1 (RPS6KB1) as a potential binding substrate for PPM1H

**Figure 3:**
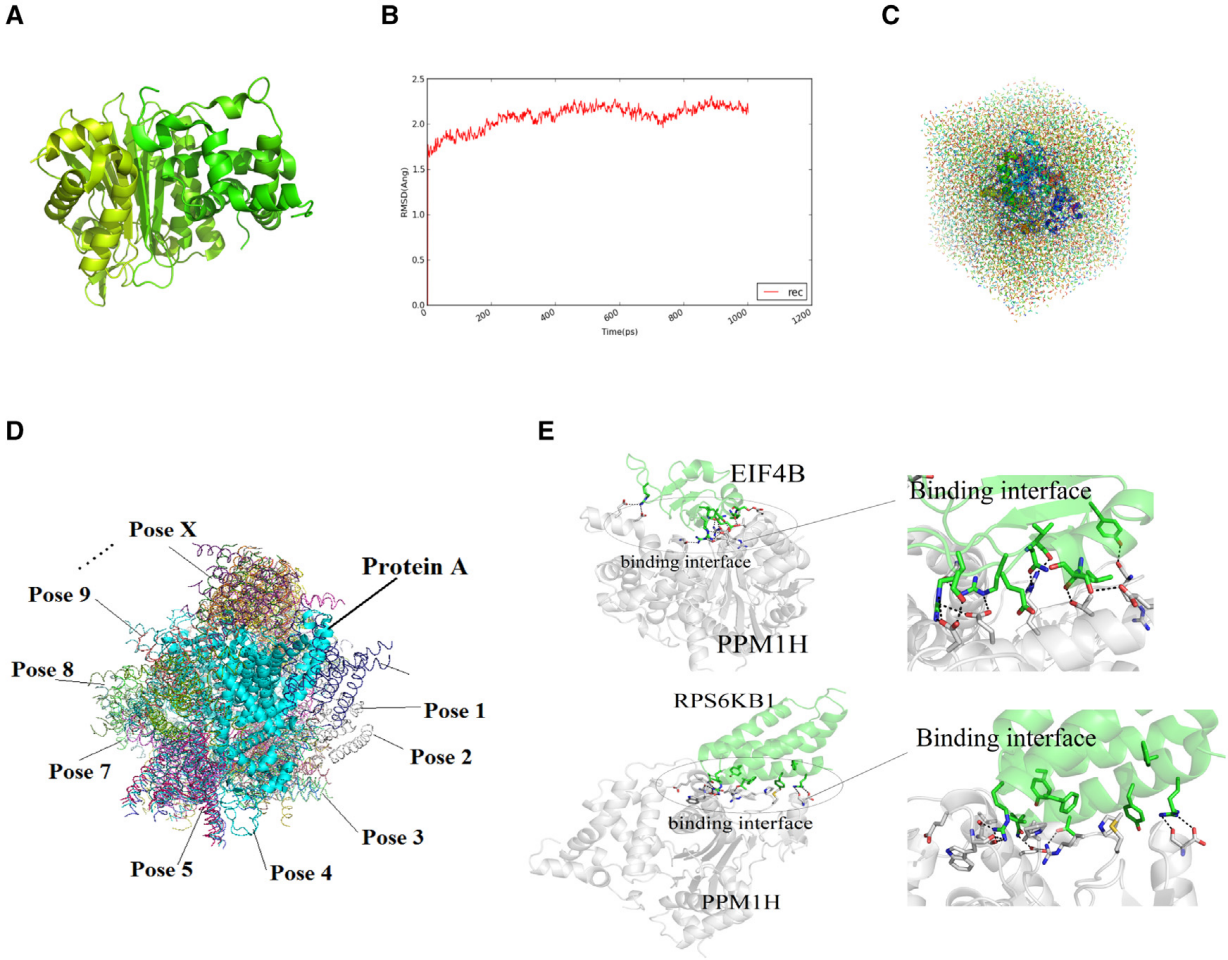
The computer simulates the PPM1H spatial simulation configuration and the docking mode of PPM1H with EIF4B and RPS6KB1 (A) Computer simulation PPM1H spatial structure pattern diagram. (B) RMSD value detection of the PPM1H spatial structure model. (C) The Tip3P water box model analyzes the molecular dynamics trajectory of PPM1H. (D) The optimal docking phase of PPM1H. (E) Docking mode and the docking interface of PPM1H, EIF4B, and RPS6KB1.

The crystal structure of the SMAD1 protein and the structure derived from the amino acid sequence of PPM1H were searched in the RCSB PDB database (Table S2). The PDB: 4JND, 4DS8, and 2ISN homologous sequences were used as modeling templates (the similarity was 20.26%, 31.98%, and 23.66%, respectively). A multi-template protein modeling method was adopted by the Modeler software to derive a 3D structure prediction of the PPM1H protein. Preliminary molecular dynamics optimization was performed by first modeling the PPM1H results. The same method was used for AMBER14, of which the steric hindrance between amino acids and the unreasonable localized regions of amino acids in modeling were eliminated. An optimized, more reliable protein structure was obtained (Figure 3A). After modeling dynamics optimization, the fluctuation of the RMSD was less than 0.5 Å based on the RMSD of the skeleton atoms in the system after 400 ps, indicating that the structure of the PPM1H model had reached an equilibrium (Figure 3B). In addition, the result of the computer simulation of the average node structure of the molecular dynamics trajectory of the target protein in the Tip3P water box model showed that the PPM1H model constructed above was structurally stable (Figure 3C). In our study, we used a computer model to simulate the biological environment and employed intermolecular interaction and protein stability characteristics to predict the spatial structure of PPM1H, especially of its active region.

We screened potential substrates for binding to PPM1H through computer simulation and mainly included substrates with serine/threonine in their active center for activation.^28^^,^^29^^,^^30^ On the other hand, we hypothesized that there were potential PPM1H substrates in the phosphatidylinositol 3-kinase (PI3K)/Akt and BMP/transforming growth factor β (TGF-β) pathways, which might affect the progression of HCC.

The potential PPM1H substrates in the PI3K/Akt and BMP/TGF-β pathways were screened by computer-simulated protein docking experiments. All protein information in both pathways was obtained from the Kyoto Encyclopedia of Genes and Genomes (KEGG) Pathway database. Afterward, the RCSB PDB structure information for the pathway proteins was downloaded. The Rosetta software was used to analysis the 113 proteins contained in the pathways, and the protein-protein molecules were analyzed separately. The closest clustering of nearly 10,000 protein docking phases was generated, and the top 10 PPM1H docking conformation energies are shown in Table S3. The best energy-matching docking phase conformation of −26.51 kcal/mol was determined as the optimal docking result of the system (Figure 3D). Table S3 shows the energy of the docking conformation, which was then used for the docking of a total of 105 protein targets of the PI3K/Akt and BMP/TGF-β pathways. The analysis revealed that EIF4B (−25.21 kcal/mol) and RPS6KB1 (−25.31 kcal/mol) showed the most stable protein targets, according to the docking energy for PPM1H. Figure 3E showed the 3D docking structure diagram and binding interface of PPM1H with EIF4B, RPS6KB1. The PPM1H binding site with two predicted substrates was located at the enzyme activity center. The docking energy of SMAD1 and PPM1H was −19.21 kcal/mol, second only to RPS6KB1 in the BMP/TGF-β pathway. Finally, according to the computed structure of PPM1H and the analysis of the docking energy between PPM1H and potential substrates, we found 16 substrates from the PI3K/Akt and BMP/TGF-β pathways with a lower docking energy than SMAD1, among them EIF4B and RPS6KB1, with the lowest PP1MH docking and the most stable binding energy.

### PPM1H directly dephosphorylates p-RPS6KB1

**Figure 4:**
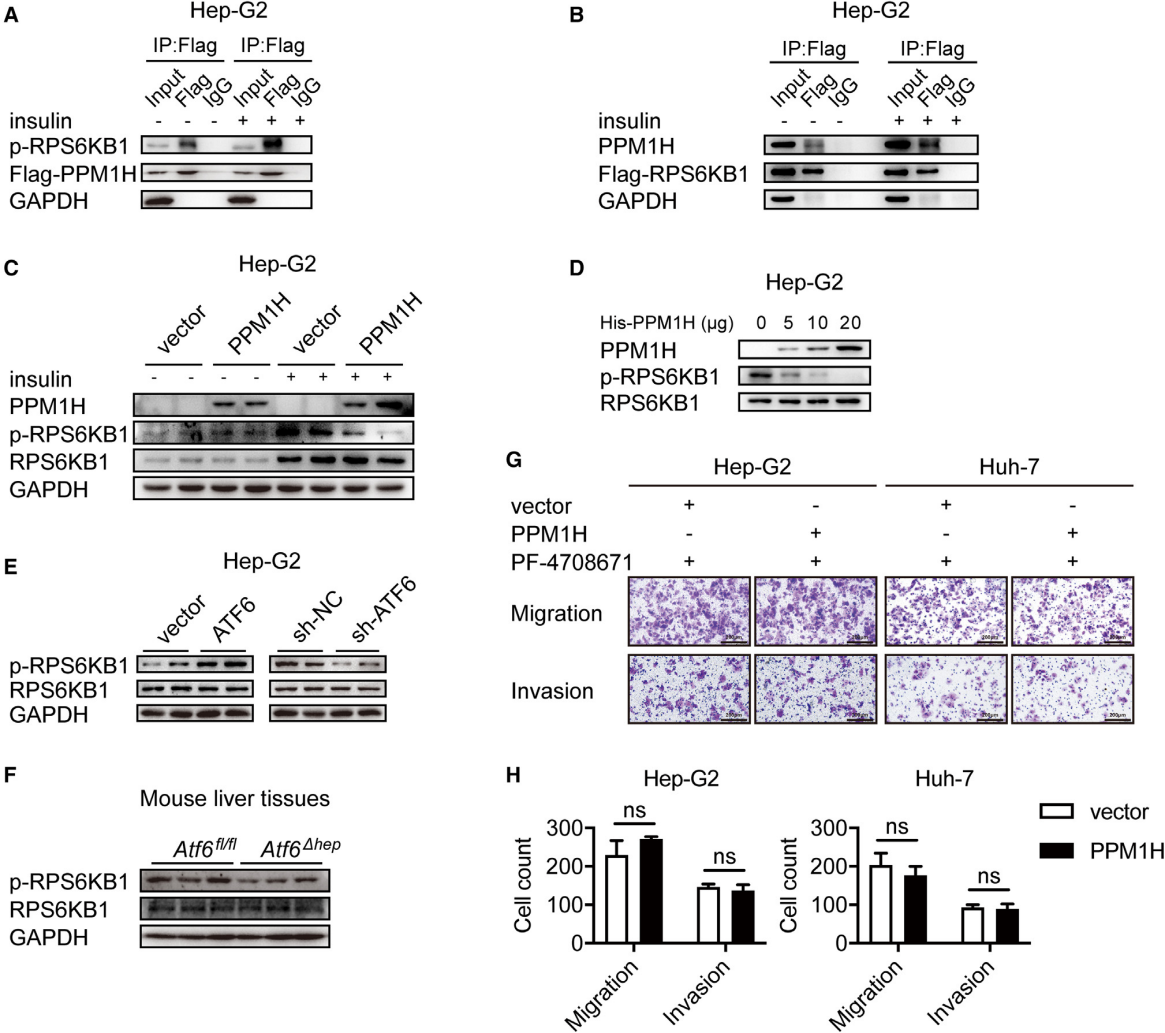
PPM1H directly dephosphorylated p-RPS6KB1 A–C) 24 h after transfection, cells were cultured in basal medium without serum overnight; insulin (10 ng/μL) was added 2 h before harvest to activate phosphorylation of RPS6KB1. (A) FLAG-PPM1H was transfected into Hep-G2 cells with or without insulin treatment, and coIP was performed to examine the level of RPS6KB1 combined with PPM1H. (B) CoIP analysis of PPM1H and RPS6KB1 in FLAG-RPS6KB1-transfected Hep-G2 cells with or without insulin treatment. (C) Western blot analysis of the indicated protein levels in PPM1H-overexpressing Hep-G2 cells with or without insulin treatment. (D) *In vitro* phosphatase assay to determine the interaction between PPM1H and p-RPS6KB1. (E and F) Western blot analysis of the indicated protein levels in vector, ATF6, sh-NC, and sh-ATF6 transfected Hep-G2 cells (E) and in liver tissues of *Atf6*^*fl/fl*^ or *Atf6*^*Δhep*^ mice. (G) The RPS6KB1 inhibitor PF-4708671 was added to cells at a concentration of 10 μM, which effectively blocked phosphorylation of p-RPS6KB1. Transwell assays revealed that the migration and invasion of PF-4708671-treated Hep-G2 and Huh-7 cells showed little difference between vector and PPM1H. Magnification, ×100. (H) Quantification of (G). Data represent the mean ± SD of three independent experiments. ns, not significant.

The docking energy of RPS6KB1 to PPM1H was −25.31 kcal/mol, lower than that of SMAD1 in the BMP/TGF-β pathway, indicating that it might be a promising PPM1H substrate. To determine whether PPM1H interacts with RPS6KB1, co-immunoprecipitation (coIP) experiments were conducted using Hep-G2 and Huh-7 cells. They were incubated with insulin (10 μg/mL), which served as an mTOR/RPS6KB1 activator. PPM1H could indeed interact with RPS6KB1, and the interaction between them was enhanced upon administration of insulin (Figures 4A, 4B, S6A, and S6B). Next, we analyzed whether p-RPS6KB1 was markedly reduced in PPM1H-overexpressing Hep-G2 and Huh-7 cells (Figures 4C and S6C), respectively. Surprisingly, knockdown of PPM1H increased the expression of p-RPS6KB1 (Figure S6D). To determine whether PPM1H directly dephosphorylates p-RPS6KB1 *in vitro*, immunoprecipitated RPS6KB1 was incubated with increasing doses of recombinant His-PPM1H protein extracted from bacteria. As a result, PPM1H dephosphorylated p-RPS6KB1 in a dose-dependent manner (Figure 4D). The PPM1H expression level is negatively regulated by ATF6, as shown previously. In Hep-G2 and Huh-7 cells overexpressing ATF6, the phosphorylation level of RPS6KB1 increased and knockdown of ATF6 inhibited p-RPS6KB1 expression (Figures 4E and S6E). The liver tissues of *Atf6*^*Δhep*^ mice also showed a lower expression level of p-RPS6KB1 than *Atf6*^*fl/fl*^ mice (Figure 4F), which suggests that ATF6 promotes phosphorylation of RPS6KB1 through PPM1H inhibition. A highly specific RPS6KB1 inhibitor, PF-4708671, was added to the culture medium at a concentration of 10 μM, which effectively inhibited phosphorylation of p-RPS6KB1.^31^ The Transwell migration and invasion assays showed that there was little difference between the PPM1H-overexpressing and control groups after PF-4708671 addition (Figure 4G), indicating that PPM1H regulates the growth of hepatoma cells through inhibition of RPS6KB1. To further validate this, two mutant plasmids expressing an inactivated RPS6KB1 mutant (T389/412A) and constitutively activated mutant (T389/412D) were constructed and co-transfected with PPM1H into Hep-G2 and Huh-7 cells, respectively. According to the western blot results, PPM1H reduced the phosphorylation level of wild-type p-RPS6KB1. The phosphorylation level of p-RPS6KB1 showed the most significant difference between T389/412A and T389/412D (Figure S6F and S6G). The migration and invasion of cells were determined through Transwell assays. Cells co-transfected with RPS6KB1-T389/412D and PPM1H showed significantly enhanced migration and invasion compared with cells co-transfected with RPS6KB1-T389/412A and PPM1H (Figure S6H). These results suggest that a PPM1H-regulated phosphorylation level of p-RPS6KB1 is associated with HCC progression.

PPM1H has been reported as the phosphatase targeting several members of the Rab GTPase family, such as Rab8A and Rab10, resulting to block of LRRK2 pathway. Abnormal activation of LRRK2 caused by gene mutation is the main cause of Parkinson’s disease.^32^ Overexpression of Rab proteins is also relative to tumor progression. Through western blot assays, we found that PPM1H could slightly inhibit the expression level of Rab5a while having barely any effect on the expression level of Rab7a (Figure S6I). Rab5a has been found to play a promoting role in the proliferation and invasion of HCC,^33^ suggesting that PPM1H may inhibit HCC progression by impairing the function of Rab5a, which needs further exploration.

### PPM1H suppresses tumor growth in mouse HCC models

**Figure 5:**
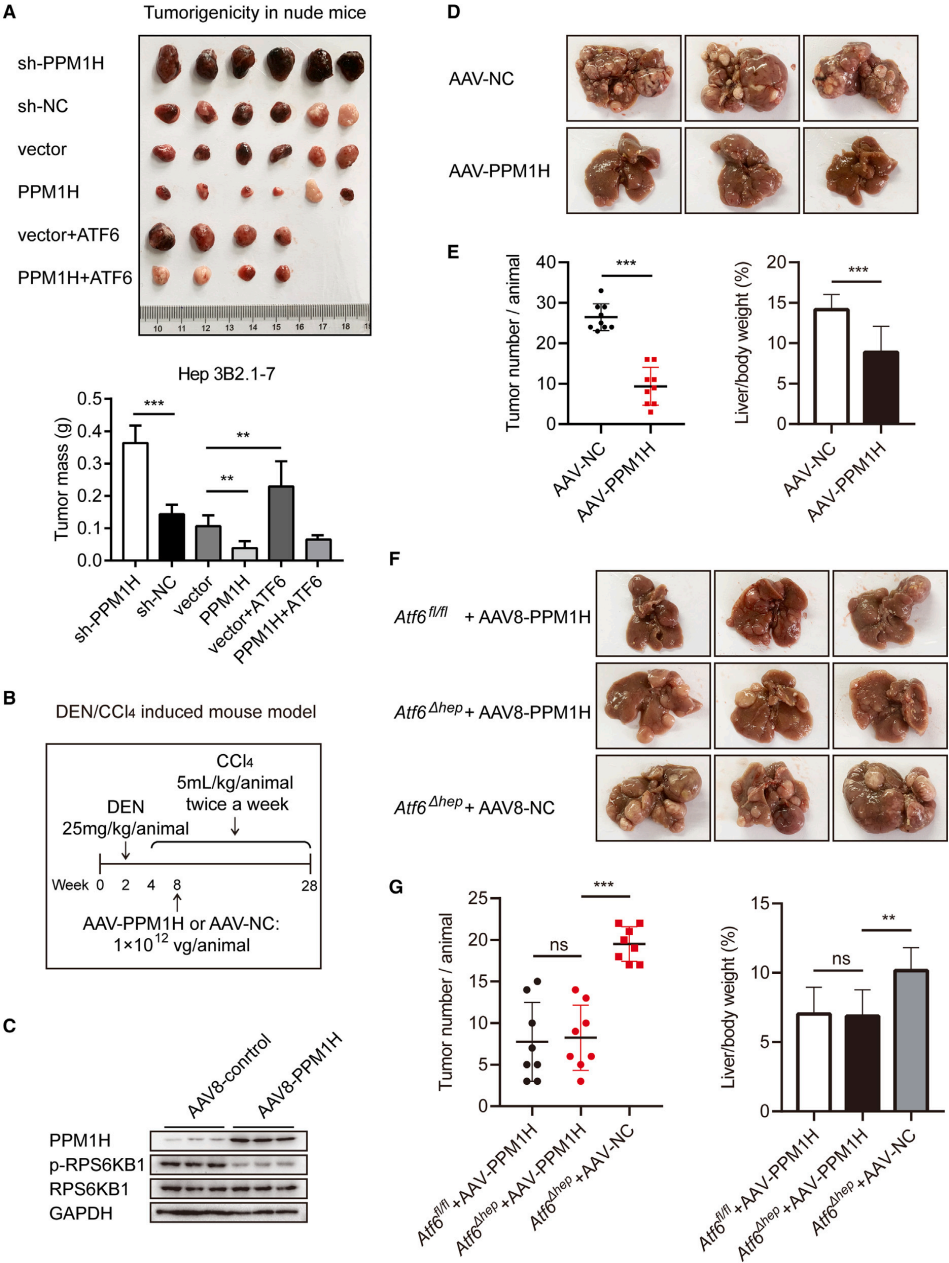
PPM1H suppressed tumor growth in mouse HCC models (A) Hep 3B2.1-7 cells infected with the indicated lentivirus were injected subcutaneously into male BALB/c nude mice; the xenograft tumors were dissected out and weighed 4 weeks later. The bottom panel shows the tumor mass. (B) Schematic of the construction of DEN/CCl_4_-induced HCC mouse models. (C) Livers of WT C57BL/6 mice were extracted 2 weeks after injection of adeno-associated virus AAV8-control or AAV8-PPM1H to perform western blot assays of PPM1H and p-RPS6KB1 protein expression. (D) Livers of DEN/CCl_4_-treated WT C57BL/6 mice injected with AAV8-control or AAV8-PPM1H (n = 9 per group). (E) The tumor numbers and ratio of liver weight to body weight were determined. (F) Livers of DEN/CCl_4_-treated *Atf6*^*fl/fl*^ and *Atf6*^*Δhep*^ mice injected with the indicated AAV8 virus (n = 8 per group). (G) The tumor number and ratio of liver weight to body weight were determined. Data represent the mean ± SD. ∗p < 0.05, ∗∗p < 0.01, ∗∗∗p < 0.001.

According to the ATCC database, Hep-G2 cells are not tumorigenic in immunosuppressed mice, while the Hep 3B2.1-7 cell line can form tumors in nude mice, which has been used widely in mouse xenograft models.^34^^,^^35^ To determine whether PPM1H inhibits tumor growth *in vivo*, we established stable PPM1H- or ATF6-overexpressing and PPM1H knockdown Hep 3B2.1-7 cells. The stable cell lines were then injected subcutaneously into male BALB/c nude mice. As shown in Figures 5A and S7A, knockdown of PPM1H obviously enhanced the tumorigenicity and tumor growth of Hep 3B2.1-7 cells, while PPM1H overexpression in Hep 3B2.1-7 cells formed smaller xenogeneic tumors than in the control group (Figures 5A and S7A). In PPM1H-overexpressing xenograft tumor tissues, the expression level of E-cadherin was increased and of N-cadherin was reduced, while knockdown of PPM1H inhibited the expression level of E-cadherin and improved N-cadherin expression (Figure S7B). Moreover, ATF6 promoted the tumorigenicity of Hep 3B2.1-7 cells, and this could be partially neutralized after ATF6-overexpressing Hep 3B2.1-7 cells were co-infected with a PPM1H lentivirus. PPM1H-overexpressing cells co-infected with ATF6 displayed a little bit larger xenogeneic tumor size than cells co-infected with a control lentivirus; however, the results were statistically not significantly different (Figures 5A and S7A). These results demonstrate that PPM1H reduces the tumorigenicity of hepatoma cells.

A diethylnitrosamine (DEN)/CCl_4_-induced HCC mouse model was constructed to further investigate the influence of PPM1H on HCC tumorigenesis. As shown in Figure 5B, either wild-type or *Atf6*^*Δhep*^ C57BL/6 mice were used for induction of HCC by injection of DEN and CCl_4_. In addition, the adeno-associated virus AAV8-PPM1H or AAV8-control virus was injected into the mouse tail vein 8 weeks after birth. After AAV8-PPM1H injection, PPM1H was overexpressed, and the phosphorylation level of RPS6KB1 was reduced, with both confirmed by western blot analysis (Figure 5C). Expression of cyclin A2 and cyclin B1 can be activated by RPS6KB1.^36^ In the livers of PPM1H-overexpressing mice, the mRNA expression of these two downstream genes was also down-regulated (Figure S7C), and the mRNA expression level of GRP78 and CHOP had barely changed in liver tissues of mice injected with AAV8-PPM1H (Figure S7D). In the induced HCC mouse models, there were less tumor nodules in the livers of *Ppm1h-*mice compared with the control group (Figures 5D and 5E), and the ratio of liver weight to body weight was also reduced (Figure 5E). The induced HCC mouse model was also constructed in *Atf6*^*fl/fl*^ and *Atf6*^*Δhep*^ mice. Among the *Atf6*^*Δhep*^ mice, PPM1H*-Atf6*^*Δhep*^ mice apparently had a reduced number of induced tumor nodules and ratio of liver weight to body weight compared with NC*-Atf6*^*Δhep*^ mice, while only a small difference between PPM1H*-Atf6*^*fl/fl*^ and PPM1H*-Atf6*^*Δhep*^ mice (Figures 5F and 5G), demonstrating that ATF6 promotes progression of HCC through regulation of PPM1H. These results revealed that PPM1H plays an inhibitory role in regulation of HCC tumorigenesis and tumor growth.

### PPM1H predicts the disease progression of HCC patients

**Figure 6:**
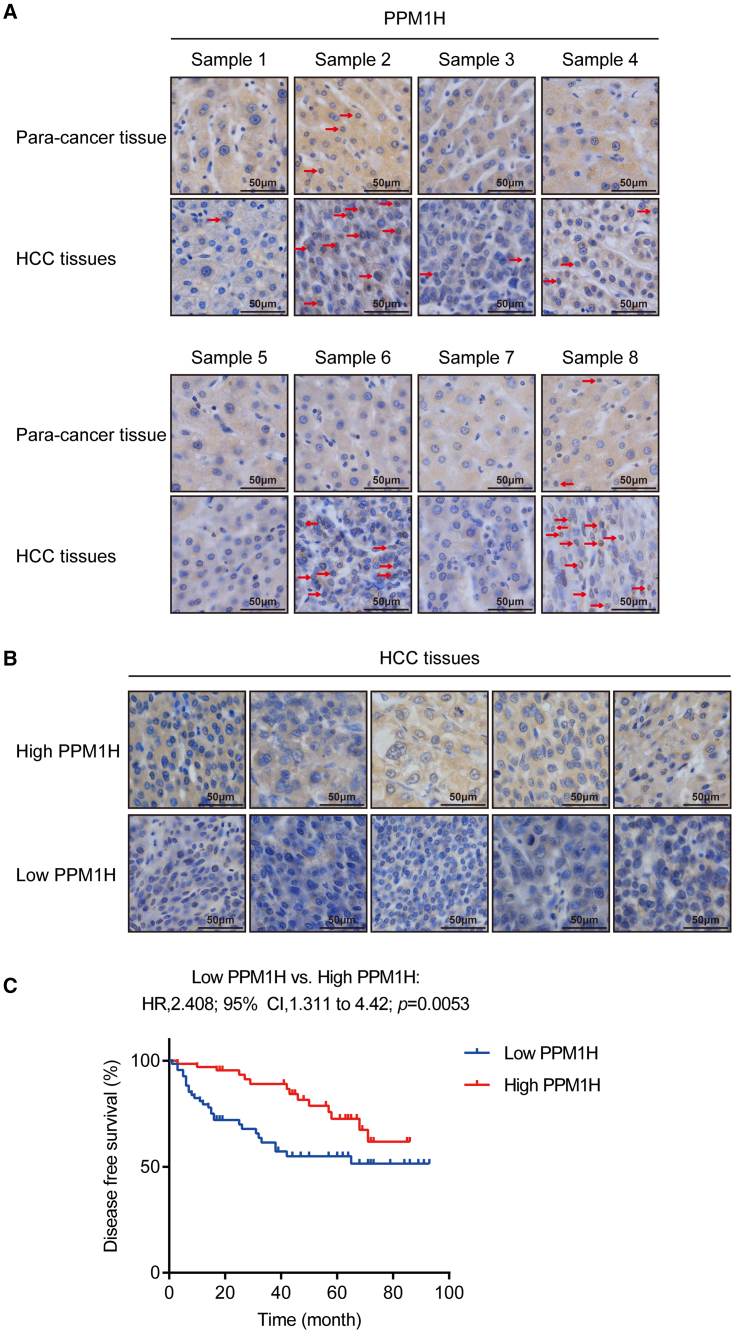
PPM1H predicted the progression of HCC patients (A) IHC analysis of PPM1H in human liver tissues. The red arrows indicate nuclear stain-positive cells. Magnification, ×400. (B) Representative IHC photos of human liver tissues with high or low PPM1H expression. Magnification, ×400. (C) Kaplan-Meier curves showing the survival of patients with low or high PPM1H expression.

To verify the PPM1H expression level of different cancer types, we analyzed breast cancer (BRCA), cholangiocarcinoma (CHOL), lung adenocarcinoma (LUAD), and liver cancer (LIHC) disclosed in The Cancer Genome Atlas (TCGA: https://www.cancer.gov/ccg/research/genome-sequencing/tcga) and Gene Expression Profiling Interactive Analysis (GEPIA: http://gepia.cancer-pku.cn/).^37^ The results showed that PPM1H expression in cancer tissues (BRCA [n = 1,085], CHOL [n = 36], and LUAD [n = 483]) was higher than in normal tissues. PPM1H was poorly expressed in LIHC (n = 369) tumor tissues, although there were no significant differences (Figure S6A). In this study, we used immunohistochemistry to detect the expression of PPM1H in 134 paraffin-embedded LIHC tissues. The results showed that PPM1H was expressed in cancer and adjacent para-cancer tissues and that the overall staining intensity of PPM1H in tumor tissues is lower than that of adjacent tissues (Figure 6A), which is consistent with the trend found in the TCGA data. Because the number of nuclear-staining-positive cells in tumor tissues was higher than that of adjacent tissues (Figure S8B), it might hint at the fact that PPM1H expression might be compartmentally increased in nuclei to exert anticancer functions, but this speculation needs to be verified.

To investigate the relationship between PPM1H expression level and clinicopathological characteristics, the 134 patients were divided into two groups according to the PPM1H expression levels in HCC tissues (Figure 6B). High PPM1H expression was associated with smaller tumor size (Table S4). Furthermore, the overall survival time of patients with a high PPM1H expression level was obviously longer than that of those expressing lower levels of PPM1H (Figure 6C), proposing that PPM1H might be a suitable predictor of HCC patient prognosis.

## Discussion

In this study, we demonstrated that ATF6 inhibits PPM1H mRNA and protein expression and decreases the phosphorylation level of PRS6KB1. Moreover, the proliferation, migration, invasion, and tumorigenicity of hepatoma cells and of induced mouse HCC cells were suppressed by PPM1H.

The serine/threonine phosphatase PPM1H was determined to be a new member of the metal-dependent protein phosphatase (PPM) family which is expressed in the cytoplasm and nucleus. The similarity of PPM1H to PPM1J (also known as PP2Cζ) in the phosphatase active region is as high as 80%,^38^ which also suggests that its enzyme activity region might be similar to other family members. PPM1H has been reported to be related to cell proliferation, differentiation, and tumor progression.^25^^,^^39^ However, the role of PPM1H may differ depending on the cancer type. It is downregulated in glioblastoma, while it was upregulated in colon cancer.^39^^,^^40^ These studies suggest that its exact mechanism of action in cancer remains controversial. In our research, we identified the suppressive role of PPM1H in hepatoma cell growth, invasion, and HCC progression. In addition, PPM1H was downregulated in human HCC tissues, and its low expression predicted a poor prognosis for HCC patients.

RPS6KB1 (also known as p70S6K or S6K1) is a serine/threonine kinase that can be activated by the PI3K/mammalian target of rapamycin (mTOR) signaling pathway. RPS6KB1 plays an important role in regulating glucose metabolism,^41^ autophagy,^42^ cell proliferation, and survival.^43^ Recent studies found that p-RPS6KB1 is upregulated in prostate cancer and that it plays an essential role in sensitization of radiation therapy.^44^ In brain tumors, correlated RPS6KB1 and hypoxia-responsive genes are upregulated, and RPS6KB1 is associated with a poor prognosis for these patients.^45^ In BRCA, RPS6KB1 phosphorylates and activates estrogen receptor α (ERα), which, in turn, enhances transcription of RPS6KB1, leading to proliferation of BRCA cells.^46^ RPS6KB1 is also upregulated in human HCC tissues compared with adjacent non-cancerous tissues and predicts a poor prognosis for these HCC patients.^47^ In our study, p-RPS6KB1 could be directly dephosphorylated by PPM1H in hepatoma cells. Phosphorylation of RPS6KB1 activated the cell-cycle-regulatory proteins cyclin A2 and cyclin B1, which then promoted progression of HCC. This implies that PPM1H might suppress HCC development through negative regulation of RPS6KB1, which affects cell cycle modulation. Insulin was added to the cells to simulate a state of highly activated p-RPS6KB1, after which we could observe an enhanced interaction between PPM1H and RPS6KB1. This points to the fact that PPM1H is more effective under conditions where the RPS6KB1 pathway is highly activated.

The phosphatase activity of PPM1H inhibits the transduction of signaling pathways involved in cancer progression. The recombinant PPM1H can directly act on the SMAD active domain and dephosphorylate the phosphorylated SMAD1/5/8.^25^ In this study, PPM1H inhibited phosphorylation of SMAD1 and RPS6KB1 in hepatoma cells. Surprisingly, a suppressive effect of PPM1H remained after the SMAD1 signaling pathway was blocked, while RPS6KB1 blocking greatly impaired the influence of PPM1H on cell migration and invasion. This suggested that RPS6KB1 plays a more important role in modulation of cell growth and invasion under PPM1H regulation.

Protein-protein docking prediction by computational methods is an important complement to experimental detection of complex structures. Virtual screening based on target structure is used in protein-small molecule docking, which has been used widely for screening of small-molecule drugs. The active ingredients and ligands are predicted by kinetic evaluation of drug similarity and drug action of substrates, which finally provides a scientific basis for exploring the mechanism of LIHC treatment.^48^^,^^49^^,^^50^ In this study, we successfully verified the effect of PPM1H on p-RPS6KB1 *in vivo* and *in vitro*. However, the model is still suffering from certain gaps in the protein interaction maps because of the fact that the network of protein interactions is extremely complex; thus, further analysis might identify more binding candidates of PPM1H. For our analysis, we focused only on the one-on-one combination of PPM1H and potential substrates. The computer simulation experiment was used as a prediction tool for exploring phosphorylation substrates. At present, the influence of PPM1H on other candidate substrates, like EIF4B, must be further explored. We will continue to identify and characterize these candidate substrates to find new targets for PPM1H in LIHC.

Overexpression of ATF6 had little impact on promoter activity or PPM1H mRNA degradation, which points to the fact that ATF6 might influence PPM1H expression through some other ways, like enhancers, which were not found in this study. Follow-up studies, such as chromatin IP (ChIP) sequencing, will be carried out to unearth the regulation mechanisms of ATF6 and PPM1H.

Immunohistochemical analysis of PPM1H expression in liver tissues of HCC patients shows that the PPM1H staining intensity in tumor tissues is somewhat lower than that of adjacent non-tumor tissues, which is consistent with the results of the TCGA analysis. However, the TCGA analysis also shows that PPM1H expression is higher in tumors of BRCA, CHOL, and LUAD than in adjacent tissues. In view of the widely distributed sample coverage population, no significant difference was shown in LIHC tissues. In future studies, we should expand HCC patient sample size.

In conclusion, we determined that mRNA and protein expression of PPM1H were inhibited by ATF6. PPM1H suppressed growth, invasion, and tumorigenicity of hepatoma cells. In HCC patients, low expression of PPM1H was associated with a poor prognosis. We discovered RPS6KB1 as a new PPM1H dephosphorylation substrate, which might play an important role in regulation of hepatoma cell development. To some extent, this study revealed the molecular mechanisms underlying HCC development, and the ATF6/PPM1H/RPS6KB1 axis may provide more candidates for therapeutic HCC targets.

## Materials and methods

Additional materials and methods can be found in the Supplemental materials and methods.

### Cell culture

The Hep-G2 (RRID: CVCL_0027), Huh-7 (RRID: CVCL_0336), and Hep 3B2.1-7 (RRID: CVCL_0326) cell lines were purchased from the National Infrastructure of Cell Line Resource (also known as Cell Culture Center of the Chinese Academy of Medical Sciences). Huh-7 and Hep 3B2.1-7 are HCC cell lines, while Hep-G2 is a hepatoblastoma-derived cell line. All human cell lines have been authenticated using short tandem repeat (STR) profiling within the last 3 years. They were cultured in Dulbecco’s modified Eagle’s medium and minimum essential medium, respectively (Gibco Life Technologies) containing 10% fetal bovine serum (FBS; Gibco Life Technologies) and maintained at 37°C in a moist atmosphere with 5% CO_2_. All experiments were performed with mycoplasma-free cells.

### Plasmids and transfection

ATF6, ATF6(1–7), PPM1H, and RPS6KB1 coding sequences were inserted into Lv-ef1a-IRES(internal ribosome entry site)-GFP or p3XFLAG-CMV(cytomegalovirus)-14 vectors to construct the overexpression plasmids. shATF6 and shPPM1H sequences were inserted into pGPU6/GFP/Neo vectors to establish the knockdown plasmids (Generay Biotechnology, Shanghai, China). PPM1H promoters were inserted into pGL3-basic to construct luciferase reporter plasmids. The target plasmid of interest was transfected into cells using Lipofectamine 3000 reagent (Invitrogen, USA).

### Antibodies

Antibodies against ATF6 (polyclonal rabbit, catalog number DF6009), RPS6KB1 (polyclonal rabbit, catalog number AF6226), p-RPS6KB1 (phospho-RPS6KB1, Thr389/Thr412; polyclonal rabbit, catalog number AF3228), and the FLAG tag (polyclonal rabbit, catalog number T0053) were purchased from Affinity (OH, USA). Antibodies against E-cadherin (monoclonal mouse, catalog number ab231303) and N-cadherin (monoclonal rabbit, catalog number ab76011) were purchased from Abcam (Cambridge, UK). Antibodies against p-SMAD1 (phospho-SMAD1, Ser463/465; polyclonal rabbit, catalog number 13820T) and the FLAG tag (polyclonal mouse, catalog number 8146) were purchased from Cell Signaling Technology (MA, USA). The anti-PPM1H (polyclonal rabbit, catalog number CSB-PA892345LA01HU) antibody was purchased from CUSABIO (Wuhan, China).

### Animals

The *Atf6*^*fl/fl*^ C57BL/6 mice were purchased from The Jackson Laboratory (ME, USA). *Alb*-cre C57BL/6 mice were obtained from the Shanghai Model Organisms (Shanghai, China). *Atf6*^*fl/fl*^ mice and *Alb*-cre mice were hybridized and generated offspring that were liver-specifically *Atf6* deficient (*Atf6*^*Δhep*^*)*. Conventional *Atf6* knockout (*Atf*^*KO*^) mice were obtained from *Atf6*^*fl/fl*^ and *Cmv*-cre C57BL/6 mice as described previously.^51^ Wild-type (WT) C57BL/6 mice and BALB/c nude mice were purchased from the Center of Medical Experimental Animals, Chinese Academy of Medical Sciences (Beijing, China). Because of the great sex differences in the incidence of LIHC, for this study, only male mice were chosen for the experiments. Animal feeding and experimental protocols were approved by the Animal Care and Use Committees and Ethics Committee of the Institute of Basic Medical Sciences, Chinese Academy of Medical Sciences.

### Human subjects

134 paraffin-embedded tissues and correlated basic information of HCC patients were collected from the Affiliated Hospital of Qingdao University for a time frame between 2010 and 2015. Written informed consent was obtained from all patients. All treatments of human subjects were approved by the ethics committee of the Affiliated Hospital of Qingdao University.

### Stable cell line establishment and nude mouse tumorigenicity assay

Lv-ATF6, Lv-PPM1H, or control plasmids were co-transfected with lentivirus packaging plasmids (delta8.91 and pVSVG), respectively, into HEK293T cells planted in 6-well plates. The supernatant culture medium containing the corresponding lentivirus was collected 72 h after transfection and used to infect Hep 3B2.1-7 cells planted in 6-well plates. Cells stably expressing ATF6 or PPM1H were selected by EGFP using flow cytometry. By geneticin (G418) selection of Hep 3B2.1-7 cell transiently transfected with pGPU6/GFP/Neo-sh-PPM1H plasmid, the stable sh-PPM1H cell line was established. 5 × 10^6^ cells were subcutaneously injected into male BALB/c nude mice aged 4 weeks. Xenogeneic tumors were collected 4 weeks later.

### DEN/CCl_4_-induced HCC mouse model

AAV8-PPM1H or AAV8-control were generated by Vigene Bioscience (Jinan, China). WT or *Atf6*^*Δhep*^ C57BL/6 mice were injected intraperitoneally with 25 mg/kg DEN (Sigma-Aldrich, USA) 2 weeks after birth. Then 5 mL/kg CCl_4_ (Sigma-Aldrich) was injected intraperitoneally twice a week for 24 weeks to induce HCC, and 1 × 10^12^ vector genome equivalents (vg)/animal AAV8-PPM1H or AAV8-control was injected into mice via tail vein 8 weeks after birth. 3 days after the final injection of CCl_4_, mice were sacrificed, and livers were extracted.

### Quantitative real-time PCR

Total RNA of cells or mouse liver tissues was extracted (Ultrapure RNA Kit, CWBIO, Beijing, China) and reverse transcribed (ReverTra Ace qPCR RT Master Mix with gDNA Remover, Toyobo Life Science, Shanghai, China) into cDNA, and then quantitative real-time PCR was performed using MagicSYBR Mixture (CWBIO). The primer sequence is listed in Table S5.

### Transwell migration and invasion

For cell migration assay, 2 × 10^5^ Hep-G2 or Huh-7 cells suspended in serum-free medium were directly planted in the Transwell upper chamber after transfection of target plasmids, and the chamber was kept in cell culture medium supplemented with 20% serum. For the cell invasion assay, cells were seeded in Matrigel basement membrane matrix in the Transwell chamber. After 48 h of incubation, cells were fixed with 4% neutral formaldehyde for 20 min, followed by staining with 0.1% crystal violet for 10 min and washing with PBS. Migration and invasion were observed via an inverted microscope. For RBS6KB1 or SMAD1 blocking, PF-4708671 (MedChemExpress, Shanghai, China) or LDN-193189 (MedChemExpress) was added to the culture medium at a final concentration of 10 μM and 0.5 μM, respectively.

### Western blot

Total proteins were extracted from cells or mouse liver tissues with radio immunoprecipitation assay (RIPA) lysis buffer (CWBIO) containing protease inhibitors (phenylmethanesulfonylfluoride [PMSF]; VWR, USA). Proteins of the ER were extracted using the ER Enrichment Kit for Tissues and Cultured Cells (Invent, Beijing, China). Proteins were separated via sodium dodecyl sulfate-polyacrylamide gel electrophoresis (SDS-PAGE) and transferred to polyvinylidene fluoride (PVDF) membranes (Millipore, Billerica, MA, USA). After blocking in 5% non-fat powdered milk, the membranes were incubated with specific primary antibodies at 4°C overnight, followed by horseradish peroxidase (HRP)-conjugated secondary antibody incubation at room temperature for 1 h. GAPDH acted as the housekeeping gene for total proteins and GRP78 for ER proteins. Chemiluminescence images were taken by Tanon-5200Multi (Shanghai, China) using Pierce enhanced chemiluminescence (ECL) western blotting substrate (Thermo Fisher Scientific, USA).

### CoIP

Total protein dissolved in RIPA lysis buffer (CWBIO) was incubated with specific antibodies or isotype nonspecific negative control antibodies at 4°C overnight with rotation. Protein A/G Plus-agarose (Santa Cruz Biotechnology, CA, USA) was added to incubate for another 2 h. Then the agarose beads were washed with cold PBS 3 times and centrifuged at 4°C and 3,000 rpm for 5 min each time to harvest the immunoprecipitated protein. Immunoblotting was performed to examine the proteins of interest. The agarose beads were diluted with 20% glycerol PBS containing protease inhibitors and preserved at −80°C to perform the *in vitro* phosphatase assay.

### Protein purification of prokaryotic expressed PTD-ATF6 and PPM1H

PTD is a short peptide (YGRKKRRQRRR) that mediates protein entry into cells.^24^ The PTD-ATF6 or PPM1H coding sequence was inserted into pET-28a+ plasmids. After transfer into *E. coli* strain Transetta (DE3) (Transgen Biotech, China), the recombinant proteins were induced by isopropyl β-D-thiogalactoside (IPTG), and the supernatant was subjected to Ni affinity chromatography after ultrasonic lysis of the bacterial solution (6×His-Tagged Protein Purification Kit – Soluble Protein, CWBIO). Then proteins were concentrated using an ultrafiltration centrifuge tube (Millipore) with a filter pore size of 30 kDa. The purified His-PPM1H was used to perform the *in vitro* phosphatase assay. PTD-ATF6 was applied to incubate cells at a certain concentration, and the culture medium was changed after 48 h.

### *In vitro* phosphatase assay

Immunoprecipitated p-RPS6KB1 was incubated with increasing dosages of PPM1H protein purified from bacteria at 37°C for 30 min. The mixtures were then subjected to western blotting using specific antibodies against p-RPS6KB1 or total RPS6KB1.

### PPM1H structural model construction and substrate screening

The 3D structure of PPM1H was predicted by Modeler software using homologous multi-templates (PDB: 4JND, 4DS8, 2ISN). Then the model was optimized for molecular dynamics using AMBER14 software in two steps. First, the protein was kept in a free state to maintain the minimum energy. Second, the long-range electrostatic energy was calculated using the PME method. The stability of the constructed model was evaluated based on the (skeletal atom root mean square [RMS] fluctuation) root-mean-square deviation (RMSD) value and the Tip3P water box model.

The pathway proteins of the PI3K-Akt and BMP/TGFβ docking analysis used Rosetta software. The protein in which the PPM1H is stably docked was screened based on the energy list of the PPM1H prediction model combined with each pathway protein. The spatial combined conformation was simulated by the software.

### Immunohistochemistry (IHC)

Tissue slides from HCC patients and mice were incubated with a PPM1H antibody. The IHC process was carried out by Servicebio (Wuhan, China). The positive rates of PPM1H nuclear staining in human tissues were quantified with 40 random tissues with paired HCC and cancer-adjacent tissues. Briefly, 100 nuclei were randomly selected in the 40 paired tissues, and the numbers of positively stained nuclei were recorded to calculate the positive rate of PPM1H nuclear staining.

### RNA-seq

Liver tissues of *Atf6*^*fl/fl*^ or *Atf6*^*Δhep*^ C57BL/6 mice were dissected to perform RNA-seq. Extraction of total RNA, library preparation for transcriptome sequencing, and the data analysis were conducted by Igenecode (Beijing, China). A total of 6 transcriptome datasets were submitted to the NCBI database, numbered as PRJNA752835. GATK (Genome Analysis Toolkit) program was used to analyze bam files acquired from the HISAT2 alignment program. GRCm39 was used as the alignment database. The minimal required read coverage was set as 1, while the final results were filtered by the following parameters: Qd < 2.0 (QualByDepth; the variant confidence divided by the unfiltered depth of non-reference samples), QUAL < 30.0 (a quality score associated with the inference of the given allele), SOR >3.0 (StrandOddsRatio; strand bias estimated by the symmetric odds ratio test), FS > 60.0 (phred-scaled p value using Fisher’s exact test to detect strand bias, the variation being seen on only the forward or only the reverse strand, in the reads), MQ < 40.0 (RMSMappingQuality; the RMS of the mapping quality of the reads across all samples), MQRankSum < −12.5 (MappingQualityRankSumTest; the U-based z approximation from the Mann-Whitney rank-sum test for mapping qualities, reads with reference bases vs. those with the alternate allele), ReadPosRankSum (ReadPosRankSumTest; the U-based z approximation from the Mann-Whitney rank-sum test for the distance from the end of the read for reads with the alternate allele).

### Statistical analysis

Statistical analysis was performed in GraphPad 8.0 software. The experiments were performed in triplicate, and the results were analyzed employing a paired t test as well as a Wilcoxon signed rank test to detect statistically significant differences on a p < 0.05 basis.

## Data availability

The raw RNA-seq data generated in this study are available in NCBI under accession number PRJNA752835. Other data that support the findings of this study are available from the corresponding author upon reasonable request.

## Supplemental information

Document S1. Figures S1–S8 and Tables S1 and S4

Table S2. PDB IDs of PI3K/Akt and BMP/TGF-β pathway proteins

Table S3. Docking energy between PPM1H with PI3K/Akt and BMP/TGF-β pathway proteins

Table S5. Primers for quantitative real-time PCR

Document S2. Article plus supplemental information
