# Supplementary material for: PPM1H is down-regulated by ATF6 and dephosphorylates p-RPS6KB1 to inhibit progression of hepatocellular carcinoma
Source: Mol Ther Nucleic Acids. 2023 Jun 19;33:164–79. doi: 10.1016/j.omtn.2023.06.013 (PMC10345229; doi:10.1016/j.omtn.2023.06.013)
Supplement: Document S1. Figures S1–S8 and Tables S1 and S4 [file mmc1.pdf]

OMTN, Volume *33*

Supplemental information

PPM1H is down-regulated by ATF6 and dephosphorylates p-RPS6KB1 to inhibit progression of hepatocellular carcinoma

Xiaoshuang Yang, Jianting Guo, Wei Li, Chunrui Li, Xilin Zhu, Ying Liu, and Xiaopan Wu

Figure. S1

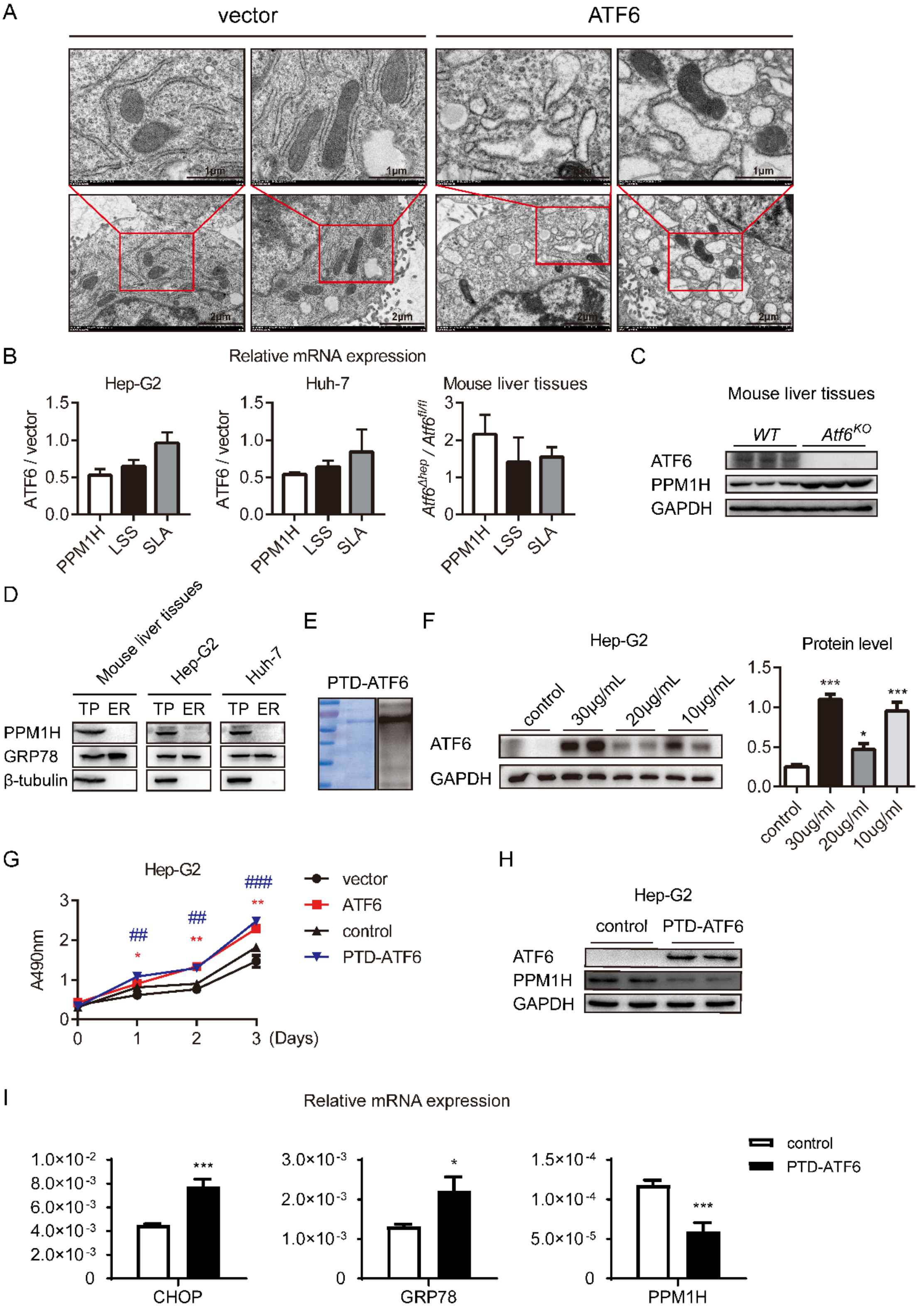

Figure. S1 ATF6 inhibits the expression of PPM1H in hepatoma cells. **A:** Hep-G2 cells were transfected with vector or ATF6 plasmids. After 48h, cells were fixed in

glutaraldehyde, followed by embedding and slicing. The morphology of ER was observed by transmission electron microscopy. The process was carried out by Servicebio (Wuhan, China). **B:** qRT-PCR was conducted to screen for potential downstream genes of ATF6 in ATF6 overexpressed Hep-G2 and Huh-7 cells and liver tissues of *Atf6*$^{\Delta hep}$ mice. **C:** Western blot analysis of ATF6 and PPM1H protein expression in mouse liver tissues. **D:** Western blot analysis of PPM1H protein expression in ER of mouse liver tissues, Hep-G2 and Huh-7 cells. β-tubulin is not located in the ER. TP, total protein. **E:** The Coomassie blue staining and western blot with anti-ATF6 antibody results revealed that recombinant protein PTD-ATF6 was obtained in vitro. **F:** The 30μg/mL of PTD-ATF6 could significantly increase the protein level of ATF6 in Hep-G2 than other concentrations, it is worth noticing that 10μg/mL and 20μg/mL PTD-ATF6 not have the measurement dependence after treatment, which probably because the concentration did not reach the threshold dose. Quantification of the protein level was shown in the right panel. **G:** MTT assay was used to detect cell proliferation after transfection of expression plasmid Flag-ATF6 or PTD-ATF6 to treat liver cancer cells. **H:** The expression of PPM1H is inhibited after treatment of HCC with recombinant protein PTD-ATF6. Quantification of the protein level was shown in the right panel. **I:** Treatment with PTD-ATF6 could promote the mRNA of GRP78 and CHOP downstream of ATF6 and reduced the expression of PPM1H. Data of qRT-PCR were normalized to GAPDH. Data represents the mean ± SD of three independent experiments. *, # are p value < 0.05; **, ## are p value < 0.01; ***, ### are p value < 0.001; ns, not significant.

Figure. S2

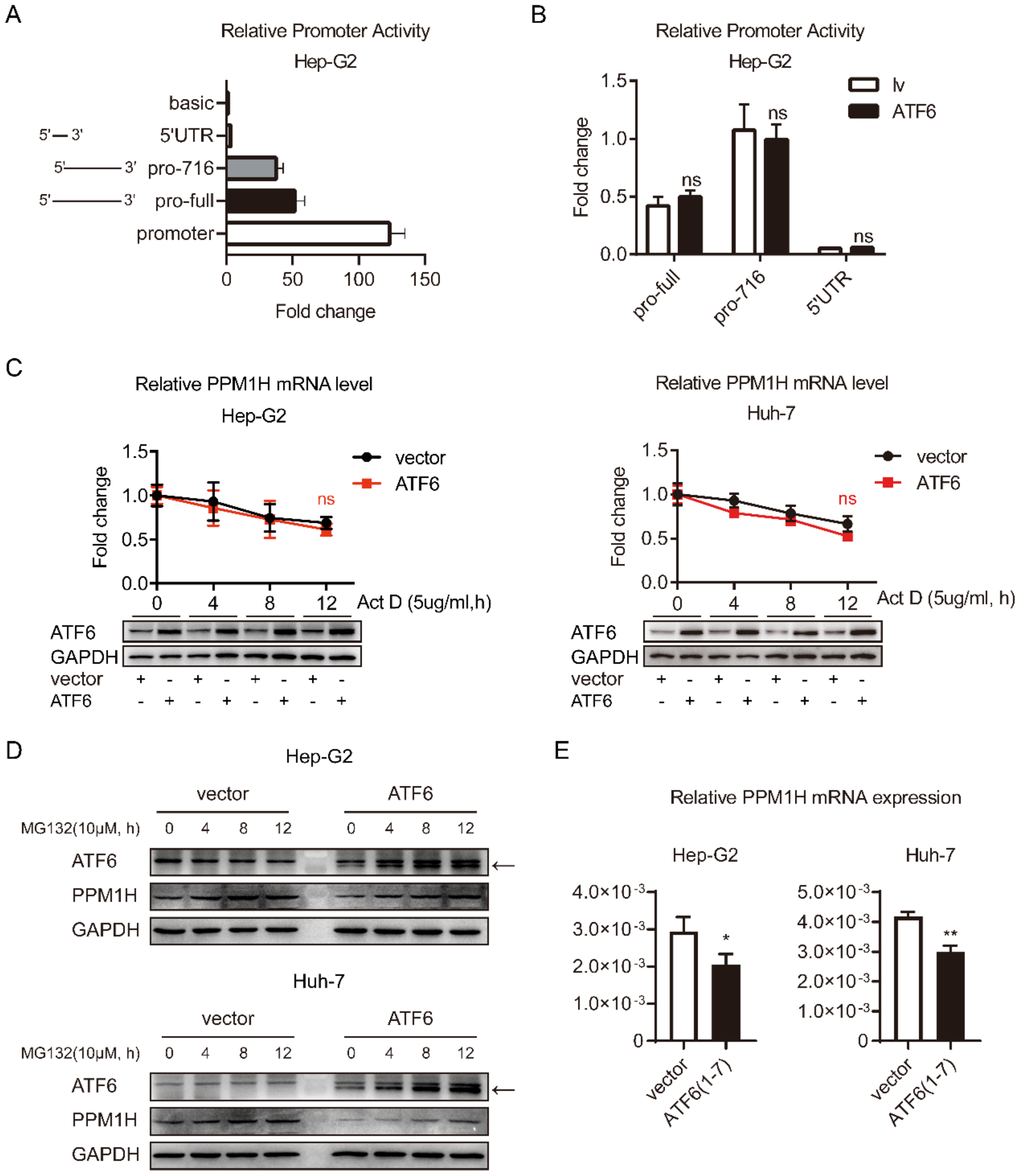

Figure. S2 ATF6 inhibits PPM1H expression trough transcription-independent manner. **A:** The luciferase activity of PPM1H promoters were examined in Hep-G2 cells. The promoter is a positive control plasmid with moderate promoter activity provided by the manufacturer, which was used to identify that the reporter gene plasmids are transcriptionally active. **B:** Co-transfection of ATF6 showed no influence

on the promoter activity of PPM1H. **C:** Actinomycin D (ActD, 5μg/mL) was added to the culture medium of Hep-G2 and Huh-7 cells 24 hours after transfection with vector or ATF6 plasmids, qRT-PCR analyzed the PPM1H mRNA level at 4h, 8h, 12h after ActD treatment. **D:** Hep-G2 and Huh-7 cells were transfected with vectors or ATF6 plasmids. After 36 hours, proteasome inhibitor MG132 was added to the cell culture medium. Western blot assays of ATF6 and PPM1H were performed after MG132 treatment for 0, 4, 8, 12h. The arrows indicated specific ATF6 blot, and the upper bands were non-specific binding of ATF6 antibody. **E:** qRT-PCR of cells transfected with ATF6(1-7) plasmids. Data of qRT-PCR were normalized to GAPDH. Dual-luciferase assay data were normalized to pRL-SV40. Data represent the mean ± SD of three independent experiments. * are p value < 0.05; ** are p value < 0.01; ns, not significant.

Figure. S3

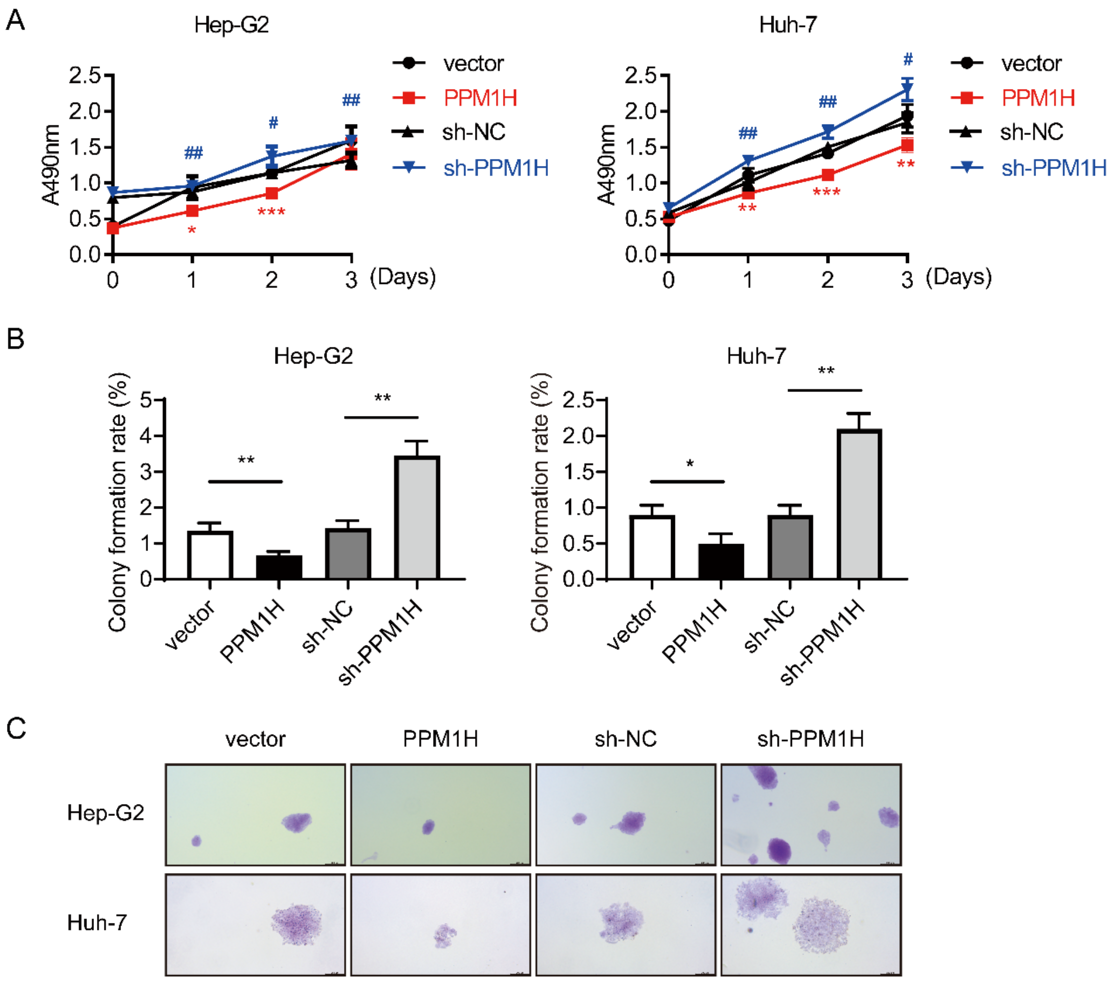

Figure. S3 PPM1H inhibited HCC cell proliferation. **A:** MTT assay analyzed the viability of Hep-G2 and Huh-7 cells transfected with vector, PPM1H, sh-NC or sh-PPM1H plasmids. **B**: Soft-agar assays of Hep-G2 and Huh-7 cells transfected with indicated plasmids. **C**: Representative images of Soft-agar assays in B. Data represent the mean ± SD of three independent experiments. * are p value < 0.05; ** are p value < 0.01; ns, not significant.

Figure. S4

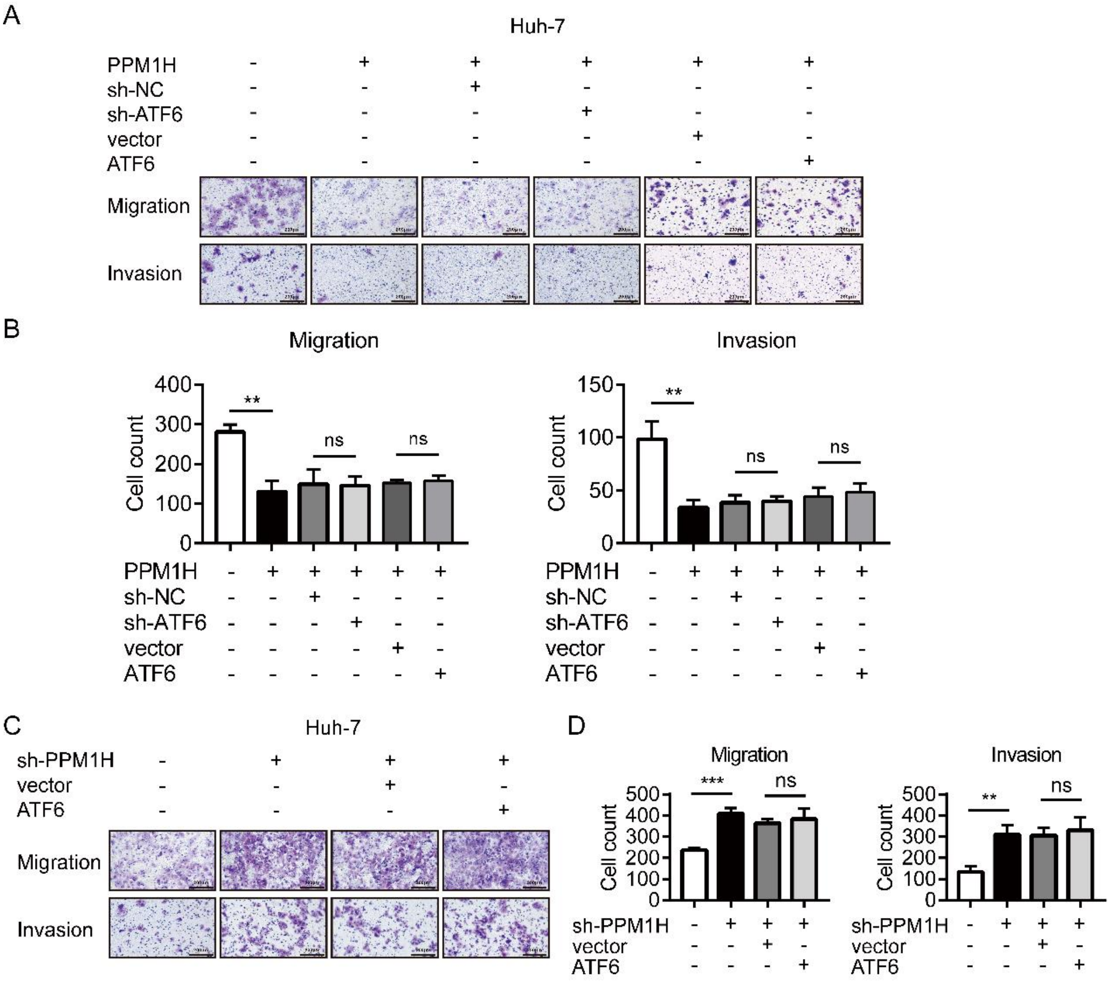

Figure. S4 PPM1H inhibited the migration and invasion of Huh-7 cells. **A:** MTT assay analyzed the cell viability. **A and C:** Transwell assays revealed the Huh-7 cells migration and invasion after transfection of indicated plasmids. Magnification × 100. **B and D**: Quantification of A and C respectively. Data represent the mean ± SD of three independent experiments. * are p value < 0.05; ** are p value < 0.01; *** are p value < 0.001; ns, not significant.

Figure. S5

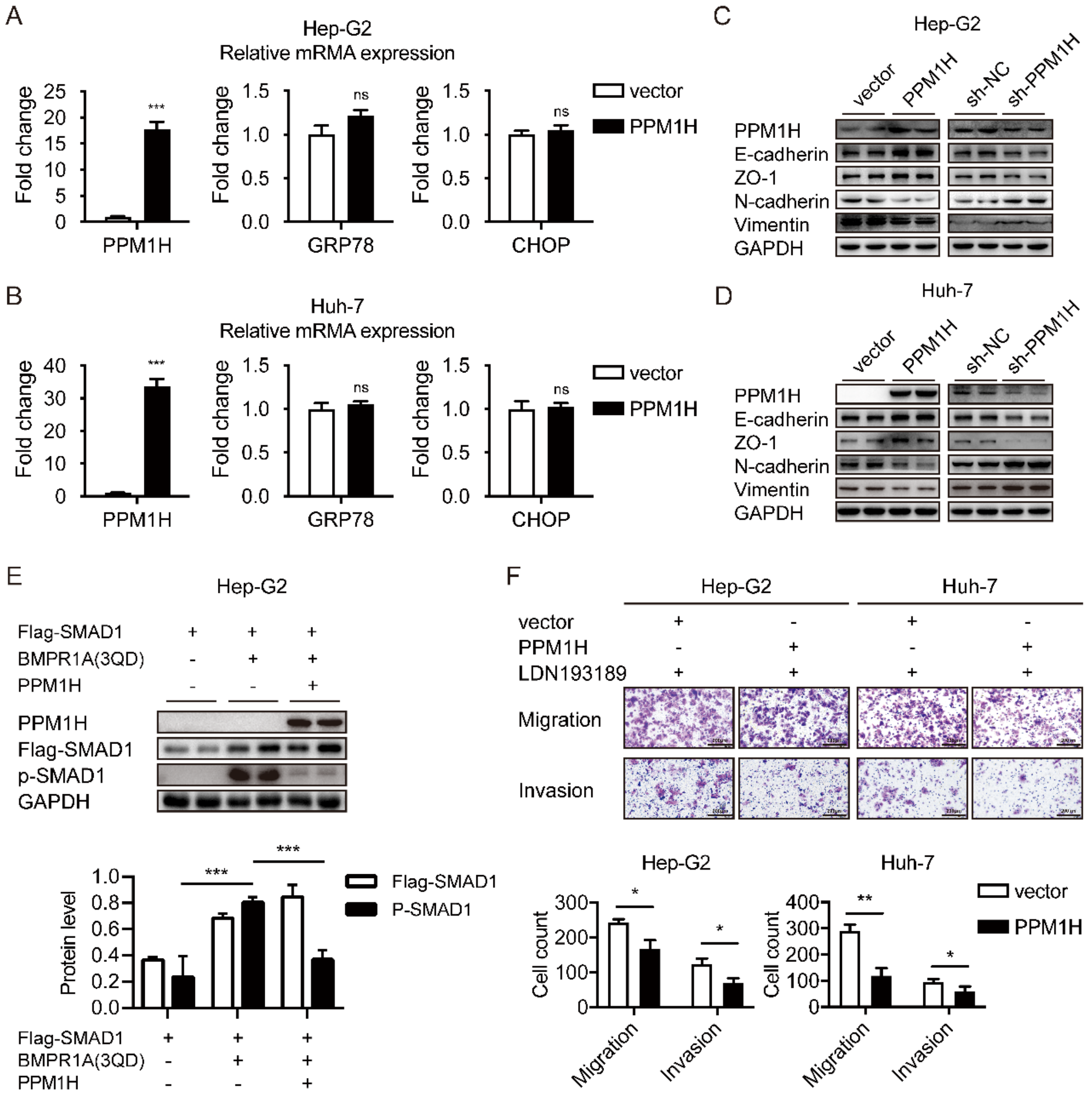

Figure. S5 PPM1H inhibited EMT and the p-SMAD1 of the BMP/TGFβ pathway. **A-B:** qRT-PCR of (A) Hep-G2 and (B) Huh-7 cells transfected with PPM1H overexpression plasmids. **C-D:** Western blot analyzed the protein expression level of EMT biomarkers in (C) Hep-G2 and (D) Huh-7 cells transfected with PPM1H overexpression or knock-down plasmids. **E:** Western blot analysis of the protein level of SMAD1 and p-SMAD1. Quantification was shown in the lower panel. **F:** Cells were treated with LDN193189 (100nM), a SMAD inhibitor blocking the BMP/SMAD1

pathway. Images and quantification of transwell migration and invasion assays in LDN193189-treated Hep-G2 and Huh-7 cells. Magnification × 100. Data represent the mean ± SD of three independent experiments. * p value < 0.05; ** p value < 0.01; *** p value < 0.001.

Figure. S6

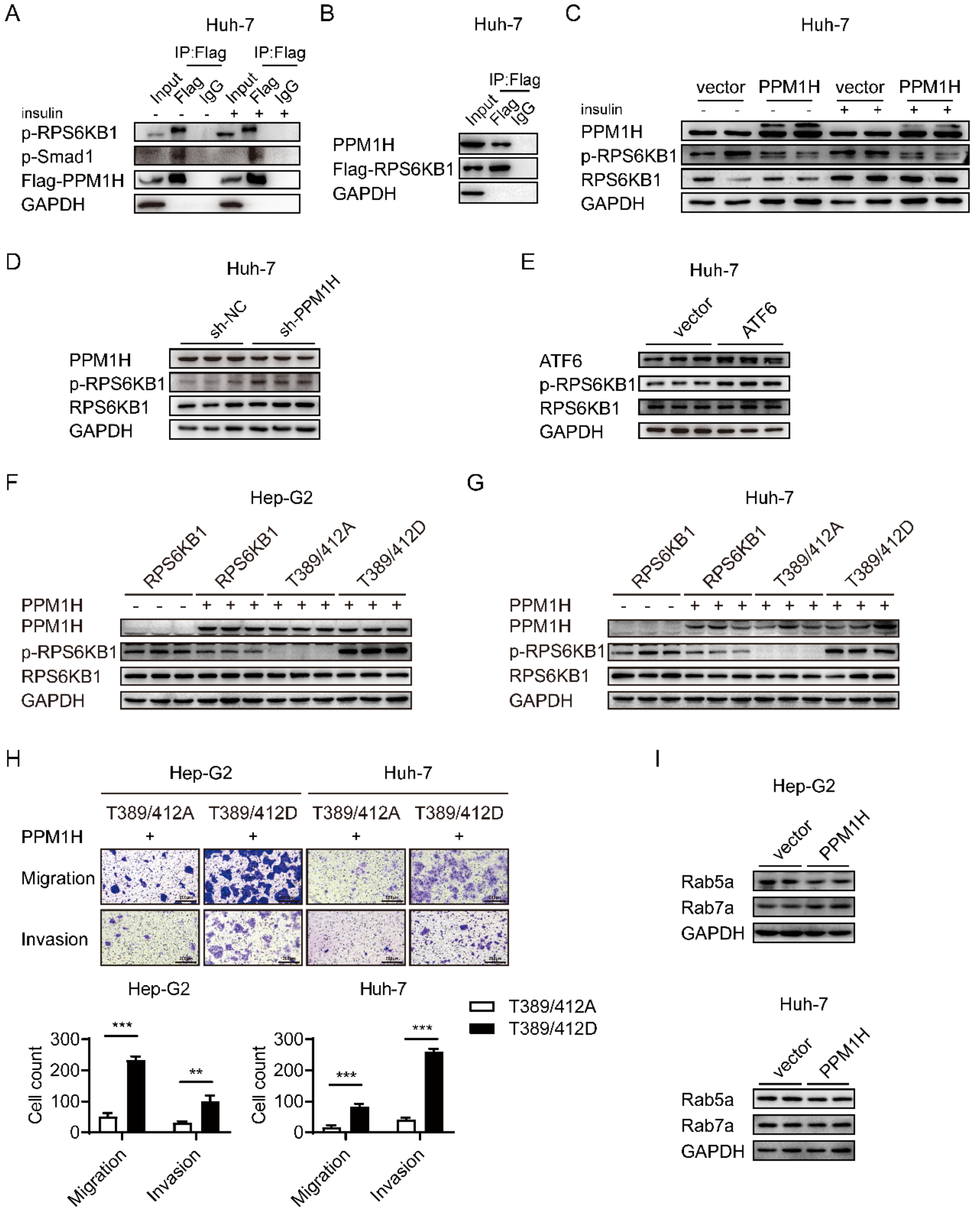

Figure. S6 PPM1H directly dephosphorylated p-RPS6KB1. **A:** Co-IP analysis of PPM1H and RPS6KB1 in Flag-PPM1H transfected Huh-7 cells with or without insulin treatment. **B:** Co-IP analysis of PPM1H and RPS6KB1 in Flag-RPS6KB1 transfected Huh-7 cells. **C:** Western blot analysis of indicated protein level in PPM1H

overexpressed Hep-G2 cells. **D:** Western blot analysis of indicated protein level in PPM1H knocked-down Huh-7 cells. **E:** Western blot analysis of the indicated protein level in ATF6 overexpressed Huh-7 cells. **F-G:** Western blot analysis of the indicated protein level in (A) Hep-G2 and (B) Huh-7 cells co-transfected with PPM1H plasmids and wildtype or mutant RPS6KB1 plasmids. **H**: PPM1H overexpressed Hep-G2 and Huh-7 cells were co-transfected with RPS6KB1 inactivated mutation plasmids (T389/412A) or continuous activated mutation plasmids (T389/412D). Representative images (upper panel) and quantification (lower panel) of transwell assays showed the migration and invasion of cells. Magnification × 100. **I:** Western blot analysis of Rab5a and Rab7a protein expression level in Hep-G2 (upper panel) and Huh-7 (lower panel) cells. Data represent the mean ± SD of three independent experiments. * p value < 0.05; ** p value < 0.01; *** p value < 0.001.

Figure. S7

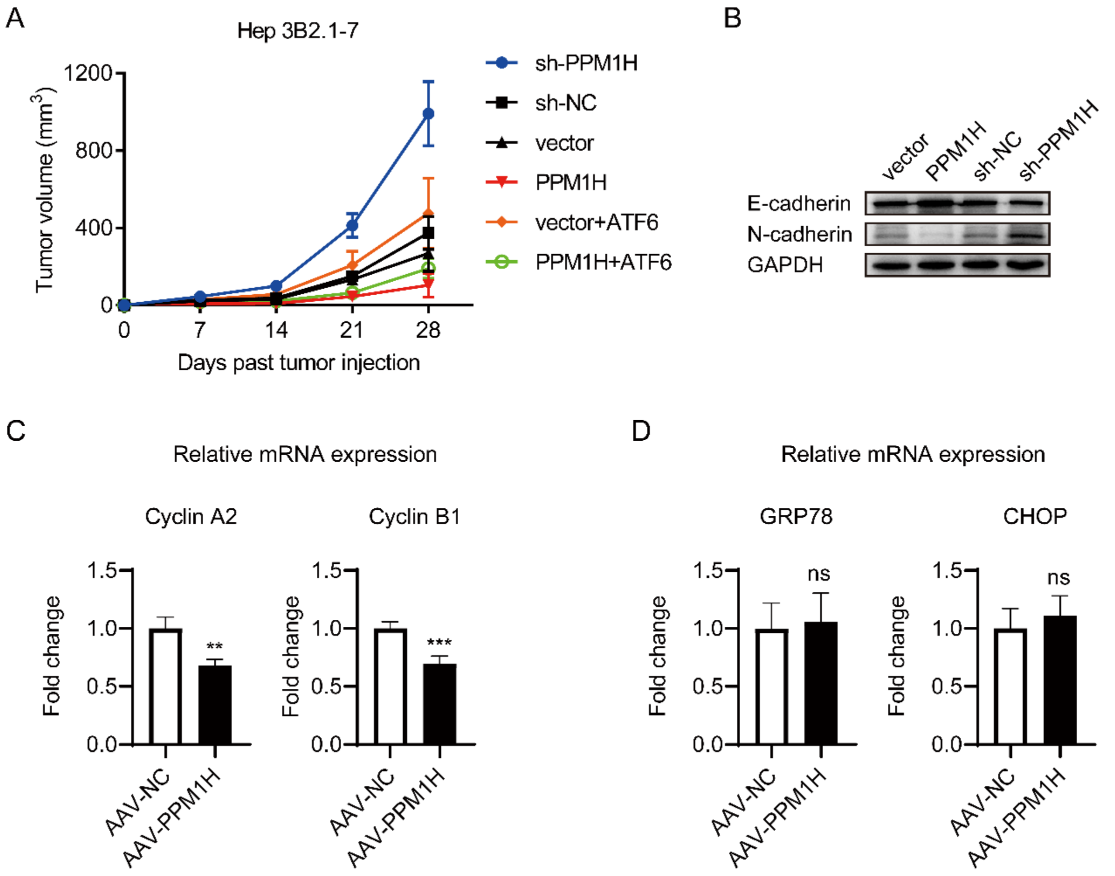

Figure. S7 PPM1H suppressed tumor growth in mouse HCC models. **A**: Hep 3B2.1-7 cells infected with indicating lentivirus were subcutaneously injected into male BALB/c nude mice. Growth curves of tumors were measured at the indicated time point. **B**: Western blot analysis of E-cadherin and N-cadherin in PPM1H overexpressed or knocked-down xenograft tumor tissues. **C**: qRT-PCR analysis of cyclin A2 and cyclin B1 which are downstream genes of RPS6KB1 in wild type C57BL/6 mice livers tissues 2 weeks after injection with AAV-NC or AAV8-PPM1H. **D**: qRT-PCR analysis of ATF6 downstream genes GRP78 and CHOP in the liver tissues of AAV-NC or AAV8-PPM1H injected mice. qRT-PCR data was normalized to GAPDH. Data represent the mean ± SD. * *p* value < 0.05; ** *p* value < 0.01; *** *p* value < 0.001.

Figure. S8

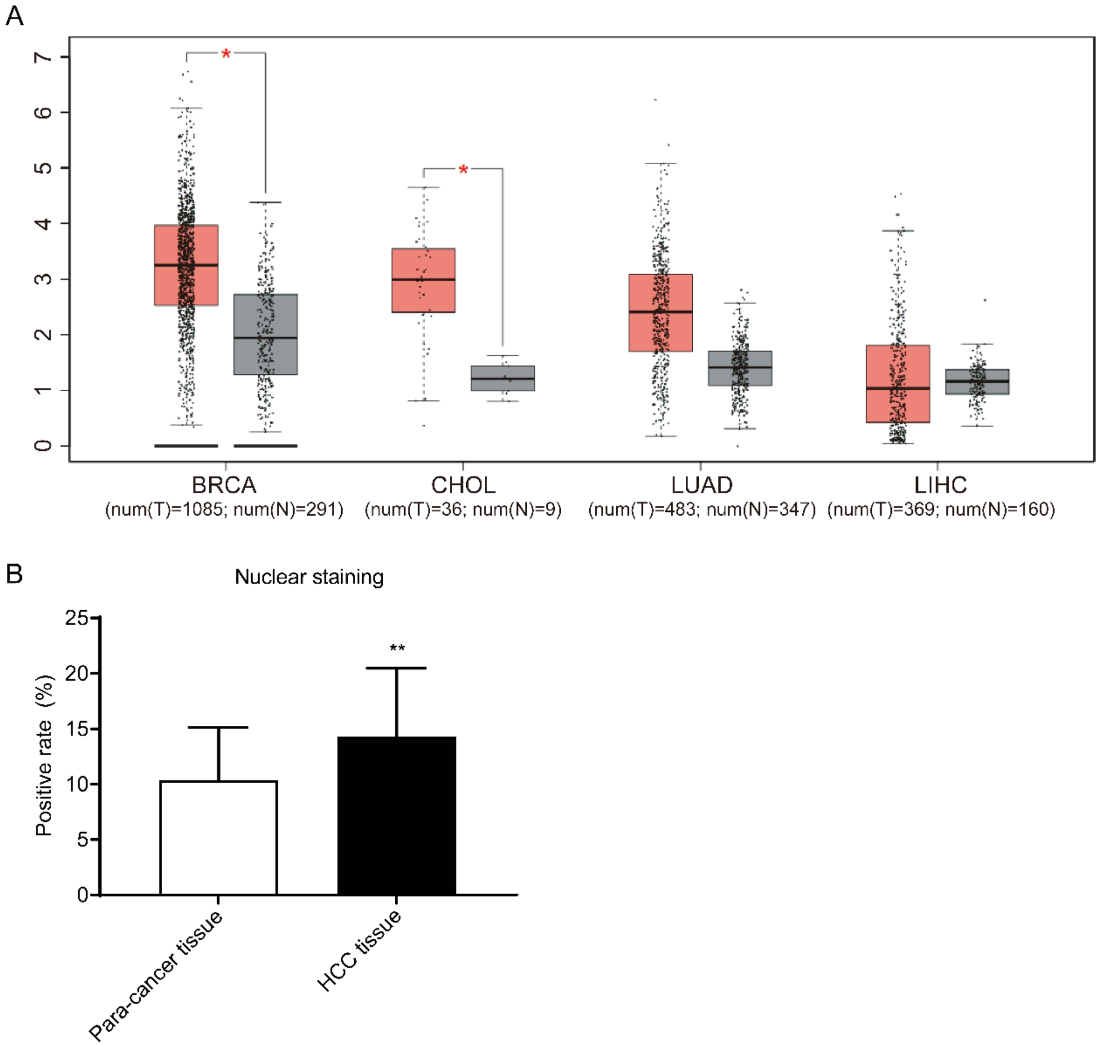

Figure. S8 PPM1H is expressed in both HCC and precancerous tissues. **A**: Expression of PPM1H in cancer samples of breast cancer (BRCA), cholangiocarcinoma (CHOL), lung adenocarcinoma (LUAD), and hepatocellular carcinoma (LIHC) disclosed in the TCGA and GEPIA database. Red represents cancer tissue samples. Gray is normal tissue sample. The results shown here are in whole based upon data generated by the TCGA (https://www.cancer.gov/tcga) and GEPIA (http://gepia.cancer-pku.cn/) research network. **B**: Positive rate of PPM1H nuclear staining. Data represent the mean ± SD. * p value < 0.05; ** p value < 0.01; *** p value < 0.001.

Table. S1 Sequencing coverage and quality statistics of the RNA-seq in mice.

Sample ID	Total number of sequenced reads	Total number of uniquely mapped reads [a]	RNA integrity number (RIN)	Ratio of all reads aligned to rRNA regions to total uniquely mapped reads (rRNA rate)	Rario of exon-mapped reads to total uniquely mapped reads (Expression Profile Efficiency)	Total number of detected transcripts with reads ≥1
ATF6-KO_060A	45531544	35073550(77.03%)	8.9	0.04%	19366998(42.54%)	31451
ATF6-KO_530A	45675306	35521604(77.77%)	8.9	0.03%	19506907(42.71%)	33369
ATF6-KO_950A	44460360	34125878(76.76%)	8.9	0.04%	18885969(42.48%)	32123
WT_420A	47203638	35247792(74.67%)	9	0.04%	19824460(42.00%)	32426
WT_430A	44812266	32468736(72.46%)	8.7	0.04%	18278569(40.79%)	30855
WT_520A	46088968	34398146(74.63%)	8.7	0.04%	20054490(43.51%)	31797

[a] GRCm39 was used as the reference genome.

Table. S2 PDB ID of PI3K/Akt and BMP/TGFβ pathway proteins.

Table. S3 Docking energy between PPM1H with PI3K/Akt and BMP/TGFβ pathway protein.

Table. S4 Correlation between PPM1H expression and clinicopathological characteristics of 134 patients with HCC.

Features		Relative PPM1H expression		*p* value
		Low	High	
Gender	Male	56	55	0.88
	Female	12	11	
Age	≤56	36	32	0.606
	>56	32	34	
Tumor size (cm)	≤5	38	48	**0.046**
	>5	27	16	
Capsular invasion	Yes	37	41	0.608
	No	23	21	
Tumor differentiation	Low	23	25	0.574
	High	44	39	
Ki-67	≤30	29	32	0.905
	>30	19	22	
Cirrhosis	Yes	53	46	0.277
	No	15	20	

Differences among variables were assessed by χ^2 or Fisher's exact χ^2 test. Bold indicates statistical differences ($p < 0.05$).

Table. S5 Primers of qRT-PCR.

Supplemental materials and methods

Dual-luciferase assay

Hep-G2 cells planted in 24-well plates were co-transfected with PPM1H luciferase reporter plasmids and ATF6 or control plasmids. The luciferase activity was analyzed 48h after transfection using Dual-Luciferase Reporter Assay System (Promega, USA).

MTT assay

Cells planted in 96-well plate were transfected with plasmids or a certain concentration of recombinant protein. 20 μL 3-(4,5-dimethylthiazol-2-yl)-2,5-diphenyltetrazolium bromide (MTT, Sigma Aldrich, USA) was added. After 4 hours of incubation, OD value at 490nm was detected to reflect the proliferation of HCC cells.

Soft-agar assay

DMEM (2×, containing 20% FBS and 2×penicillin-streptomycin) was evenly mixed with 1.2% Agarose to form the base layer containing 0.6% Agarose in a 6-well plate. Two days after transfection with indicted plasmids, cells were resuspended in DMEM containing 0.35% agarose and planted in the 6-well plate at a number of 5×10^3 cells per well. After 14 days of incubation, the colonies were fixed with 4% neutral formaldehyde for 30 minutes and stained with 0.005% crystal violet for 60 minutes. The number of colonies in each well was counted and representative pictures were taken by inverted microscope. The colony formation rate was calculated as colony numbers/ number of inoculated cells.
